# HOPS, CORVET and newly-identified Hybrid tethering complexes contribute differentially towards multiple modes of endocytosis

**DOI:** 10.1038/s41598-023-45418-3

**Published:** 2023-10-31

**Authors:** Seigo Terawaki, Filipp Vasilev, Takahito Moriwaki, Takanobu Otomo

**Affiliations:** Department of Molecular and Genetic Medicine, Kawasaki Medical School, 577 Matsushima, Kurashiki, Okayama 701-0192 Japan

**Keywords:** Membrane trafficking, Endocytosis

## Abstract

Vesicular transport driven by membrane trafficking systems conserved in eukaryotes is critical to cellular functionality and homeostasis. It is known that homotypic fusion and vacuole protein sorting (HOPS) and class C core endosomal vacuole tethering (CORVET) interact with Rab-GTPases and SNARE proteins to regulate vesicle transport, fusion, and maturation in autophagy and endocytosis pathways. In this study, we identified two novel “Hybrid” tethering complexes in mammalian cells in which one of the subunits of HOPS or CORVET is replaced with the subunit from the other. Substrates taken up by receptor-mediated endocytosis or pinocytosis were transported by distinctive pathways, and the newly identified hybrid complexes contributed to pinocytosis in the presence of HOPS, whereas receptor-mediated endocytosis was exclusively dependent on HOPS. Our study provides new insights into the molecular mechanisms of the endocytic pathway and the function of the vacuolar protein sorting-associated (VPS) protein family.

## Introduction

Intracellular degradation of biomolecules is as important and essential as biosynthetic mechanisms in maintaining cellular function and viability. Lysosomes, which play a major role in intracellular degradation^1^, contain a wide variety of hydrolytic enzymes that degrade proteins, nucleic acids, lipids, and carbohydrates as substrates and are responsible for the degradation and recycling of substrates transported from inside and outside the cell by autophagy or endocytosis^2–4^. In autophagy, a double lipid membrane called an isolation membrane expands and incorporates cytoplasmic aggregated proteins, damaged organelles, or invading bacteria into vesicles that are called autophagosomes. In endocytosis, extracellular substrates are incorporated into pits formed by invaginating plasma membranes and encapsulated in vesicles called endosomes. Both kinds of vesicles are transported to the vicinity of lysosomes where tethering molecules facilitate their fusion with the lysosomes, enabling their contents to be degraded by the hydrolytic enzymes^2,4,5^.

It is known that endocytic pathways are regulated by two homologous tethering complexes; HOPS and CORVET^6,7^. They are both composed of six vacuolar protein sorting-associated (VPS) proteins, which are evolutionarily conserved among species. HOPS and CORVET share four subunits (VPS18, VPS11, VPS16, and VPS33A, called the “class C core”), additionally have two Rab-binding subunits at both ends (VPS41 and VPS39 for HOPS and VPS8 and VPS3 for CORVET, refer to Fig. 2a), and collaborate with Rab-GTPases to facilitate the fusion of vesicles such as endosomes, autophagosomes, and lysosomes^5,6,8,9^. It is reported that genetic alterations in some *VPS* genes are associated with diseases; pathogenic variants in VPS16, VPS11, VPS41, and VPS8 cause adolescent-onset primary dystonia, autosomal-recessive leukoencephalopathy, ataxia and dystonia with retinal dystrophy and mental retardation, and multiple joint contractures and delayed motor development, respectively^7,10–14^. Furthermore, we and other groups have recently reported a new disease with mucopolysaccharidosis and sphingolipidosis-like symptoms caused by a homozygous mutation in the *VPS33A* gene^15–20^. The structure and physiological functions of HOPS and CORVET are relatively well understood^21–24^. However, considering that VPS gene defects cause a variety of phenotypes, it is unclear whether all of these VPS defect effects can be explained by HOPS or CORVET-mediated functions. In the present study, we extensively established and characterized VPS knock-out (KO) cell lines in order to compare the effects of a single defect of each VPS molecule on autophagic and endocytic pathways.

## Results

### Rab-GTPase interacting subunits VPS41 and VPS39 are required for autophagic flux

HOPS and CORVET are both composed of six vacuolar protein sorting-associated (VPS) proteins, which are evolutionarily conserved among species. HOPS and CORVET share four molecules, including VPS33A, VPS16, VPS11, and VPS18 as core subunits that are called the “class C core”. HOPS has VPS41 and VPS39, which are reported to bind to Arl8 and Rab7/Rab2 respectively, while CORVET has VPS8 and VPS3 which bind to Rab5^4,6,8,9,25–30^. To investigate the effect of the loss of these Rab-GTPase-binding tethering complex subunits on autophagy and endocytosis, we generated KO cells lacking the respective molecules in HeLa cells by the CRISPR/Cas9 system (Supplementary Fig. 1).

**Figure 1:**
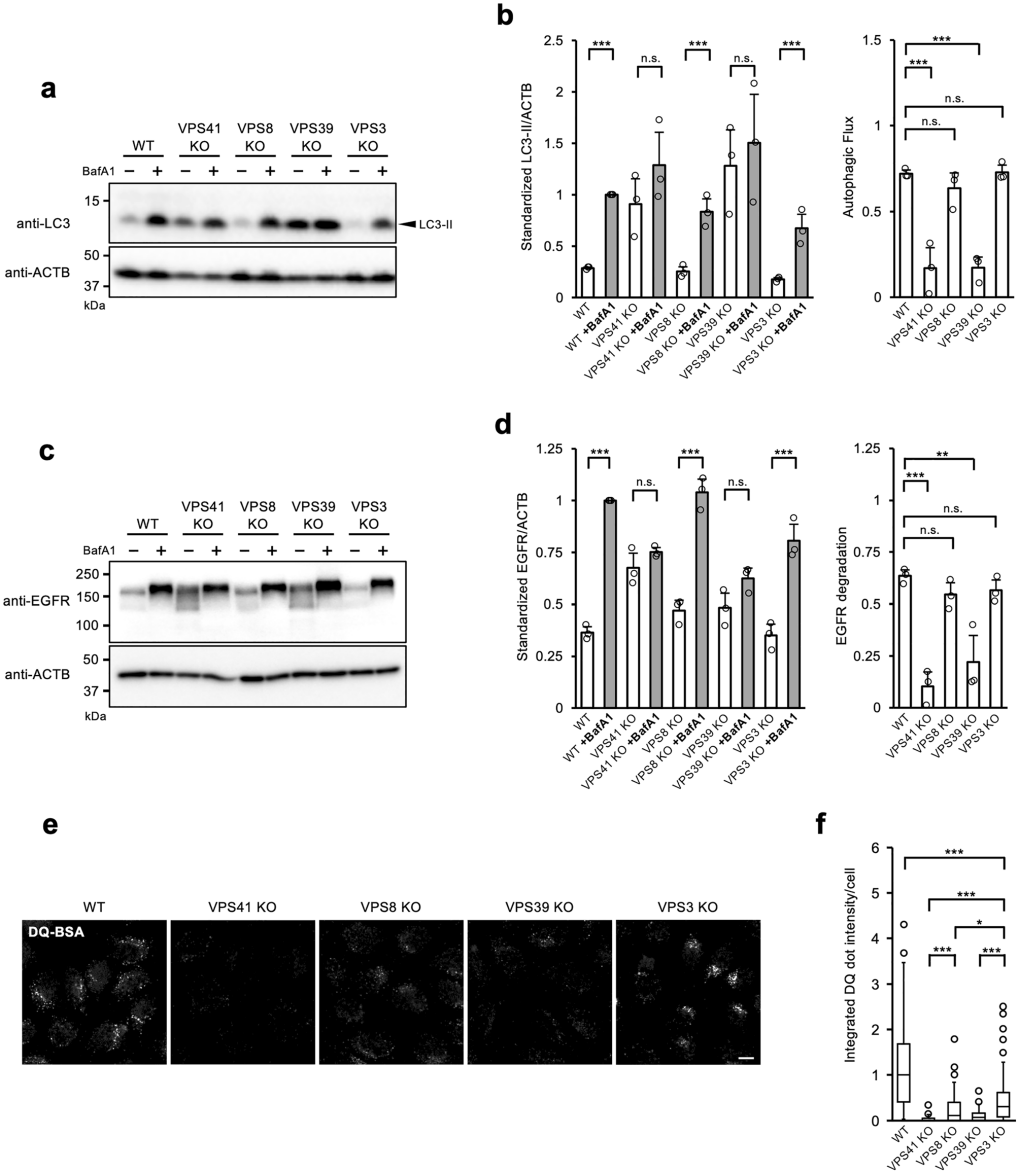
Phenotypic differences in autophagic flux and endocytosis in VPS KO cells. (**a**) LC3 flux in VPS KO HeLa-Kyoto cells. WT and VPS KO cells were cultured in the absence or presence of BafA1 for 2 h. Total cell extracts were analysed for LC3 and β-actin (ACTB) by western blotting. Representative blots are shown from 3 independent experiments. (**b**) Quantitative analysis of band intensities in panel (**a**). Intensities of LC3 divided by ACTB were normalized to BafA1-treated WT cell samples as 1. Autophagic flux shown in the right panel was calculated as follows: (LC3^BafA1+^–LC3^BafA1−^)/LC3^BafA1+^. Mean and S.D. are shown. Overlayed-circle represents each data point. Statistics were calculated by one-way ANOVA followed by post hoc Tukey’s test. (**c**) Receptor-mediated endocytosis in VPS KO HeLa-Kyoto cells. WT and VPS KO cells were stimulated with 100 ng/mL of EGF for 2 h in the absence or presence of BafA1. The whole cell lysates were analysed for EGFR and ACTB by western blotting. Representative blots are shown from 3 independent experiments. (**d**) Quantitative analysis of band intensities in panel (**c**). Mean and S.D. from 3 independent experiments are shown. EGFR degradation was determined by the following formula: (EGFR^BafA1+^–EGFR^BafA1−^)/EGFR^BafA1+^. Overlayed-circle represents each data point. Statistics were calculated by one-way ANOVA followed by post hoc Tukey’s test. (**e**) DQ Red-labelled BSA was incorporated into WT and VPS KO HeLa-Kyoto cells for 4 h and fluorescent images of activated DQ Red were acquired by confocal microscopy. A representative image from each sample is shown. *Bars* = 10 µm. (**f**) Quantitative analysis of DQ Red-BSA intensity in panel (**e**). Five images for each cell were captured, and analysed by Cell Profiler™. Integrated fluorescence intensities of DQ Red for each cell are normalized to the median value of WT and are displayed as a box-and-whisker plot. The whisker shows either the range of the data points or 1.5 IQR at maximum. Each circle represents the outlier that is outside of the whisker range. The Boxes represent the upper quartile (top line), median (middle bar), and lower quartile (bottom line), respectively. The counted cells for WT; n = 57, VPS41 KO; n = 40, VPS8 KO; n = 44, VPS39 KO; n = 50, VPS3 KO; n = 56. Statistics were calculated by the Kruskal–Wallis test followed by the Mann–Whitney U test with Holm correction. * *p* < 0.05, ** *p* < 0.01, *** *p* < 0.001, and n.s.; not significant.

Wild-type (WT) HeLa cells and KO cells of each subunit comprising HOPS or CORVET were treated with or without bafilomycin A1 (BafA1), an inhibitor of V-ATPase to stop lysosomal degradation. Total proteins were then extracted and analysed by western blotting for LC3 quantification. We found that the flux of LC3 was drastically decreased in the VPS41 and VPS39 KO cells, which are components of HOPS. This result is consistent with previous reports that HOPS binds to the SNAREs complex and promotes autophagosome-lysosome fusion^6,9,25,31,32^. On the other hand, LC3 flux in the VPS8 or VPS3 KO cells, which lack the components of CORVET was almost intact (Fig. 1a,b, Supplementary Fig. 1). These autophagic phenotypes of KO cells were also confirmed by LC3 dot flux (Supplementary Fig. 2). Thus, these results, as well as previous reports that deletion of VPS41 or VPS39 abolishes autophagic flux in either cell line, both reaffirm the important role of HOPS in autophagy progression^5,33,34^ and validate our established VPS KO cell lines^35^.

### The requirement for subunits of the tethering complex differs depending on the mode of endocytosis

In contrast to HOPS which includes Rab7/Arl8/Rab2-interacting molecules, CORVET, which can interact with Rab5 through its subunits VPS8 and VPS3, is thought to regulate early endosome fusion and dynamics^5,6,36^. Several modes of endocytosis are known, such as receptor-mediated endocytosis, macropinocytosis (hereafter described simply as “pinocytosis”), phagocytosis, and so on^3,7^. Cells use different modalities of endocytosis depending on the characteristics of substrates or biological purposes after uptake. We next investigated the functions of each VPS molecule on the different types of endocytosis including receptor-mediated endocytosis and pinocytosis.

Epidermal growth factor receptor (EGFR), which is expressed on the cell surface, is incorporated inside cells by clathrin-dependent endocytosis upon binding with their ligand EGF. Incorporated EGFR molecules are eventually degraded by the lysosomal system, although part of them is recycled to the plasma membrane^4^. We investigated the effect of VPS molecule deficiency on receptor-mediated endocytosis by assaying the degradation of EGFR. As expected from the preceding report^37^, the HOPS components VPS41 or VPS39-deficient cells showed a marked reduction in EGFR degradation compared with WT cells. Unexpectedly, the cells deficient in VPS8 or VPS3, the components of CORVET, showed EGFR degradation comparable to those of WT cells (Fig. 1c,d). These results suggest that HOPS also plays an essential role in this type of endocytosis, while CORVET is not critical.

Next, we examined the effect of VPS deficiency on pinocytosis. We evaluated the process of pinocytosis using BSA conjugated with DQ dye, such that fluorescence is activated by proteolytic cleavage of the backbone protein, BSA, in endo-lysosomes^38^. WT or the VPS KO cells were treated with DQ-BSA containing medium and DQ fluorescence was analysed by confocal microscopy to test their function on pinocytic degradation. The DQ dye was well-activated in WT cells which shows that DQ-conjugated BSA was transported to the lysosomal compartment and underwent proteolytic cleavage. In contrast, DQ signals in all tested VPS KO cell lines were significantly lower than in WT cells (Fig. 1e,f). Notably, VPS3 KO cells showed a relatively moderate reduction in the DQ signal, while it was further diminished in VPS8 KO cells than in VPS3 KO cells (Fig. 1e,f). It was expected that either deletion of VPS8 or VPS3 would theoretically cause a functional defect of CORVET and present similar results. Although VPS8 KO and VPS3 KO cells showed a comparable level of EGFR endocytosis, the loss of VPS8 showed a greater impact on pinocytosis than the loss of VPS3. This difference in pinocytosis observed between VPS8 KO and VPS3 KO cells cannot be explained merely by the defect of CORVET—and suggests the presence of an alternative, independent function of VPS3 or VPS8 or the existence of another tethering complex containing them that works in pinocytosis.

### The VPS family molecules form “Hybrid” complexes of HOPS and CORVET in mammalian cells

**Figure 2:**
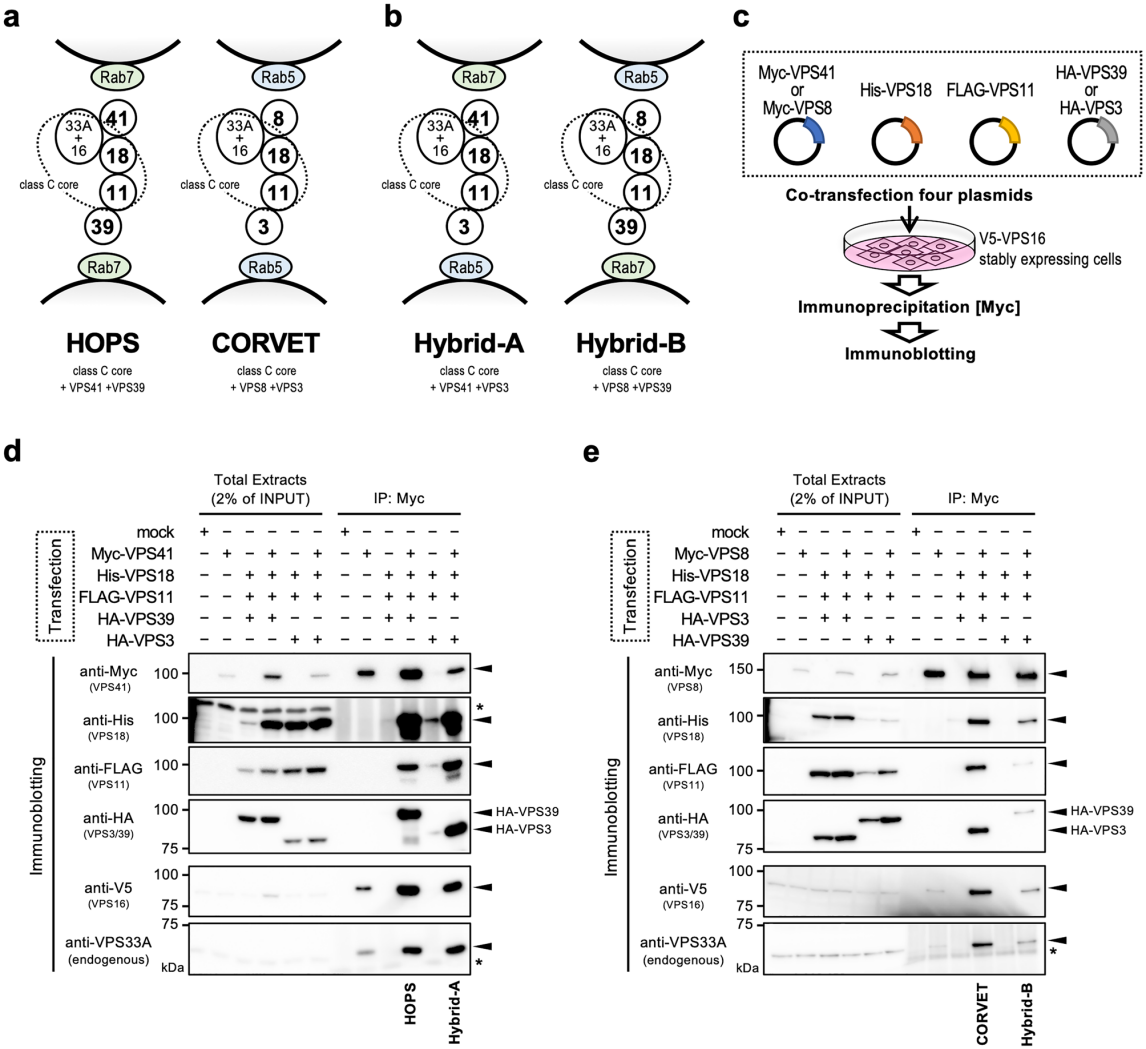
“Hybrid” complexes composed of the subunits of HOPS/CORVET. (**a**,**b**) Schematic representation of HOPS and CORVET (**a**), and non-canonical tethering complexes named Hybrid-A and Hybrid-B (**b**) interacting with Rab5- or Rab7-positive vesicles. (**c**) Experimental strategies to reconstitute and immunoprecipitate the tethering complexes with over-expressed subunits with different tags. (**d**,**e**) The HOPS/CORVET subunits which can form a complex with Myc-VPS41 (**d**) or Myc-VPS8 (**e**) were determined by immunoprecipitation (IP) analyses. A combination of the introduced plasmids for each lane is shown on the top of the blots. The left half of each panel shows input control without IP. The expected bands of each blot were indicated by arrowheads on the right side. The name of the complex corresponding to each band combination detected after IP is shown at the bottom. An asterisk (*) represents a non-specific band.

To understand the mechanism of the differential requirement of VPS8 and VPS3 for pinocytosis, we examined the possibility of the existence of unknown complexes. The class C core, composed of VPS18, VPS11, VPS16, and VPS33A, is shared between HOPS and CORVET (Fig. 2a). VPS41 and VPS8 or VPS39 and VPS3 are structurally homologous proteins and may interact similarly with the class C core, respectively^6,7,9^. We assumed two complexes composed with class C core + VPS41 + VPS3 (hereafter referred to as “Hybrid-A”), and class C core + VPS8 + VPS39 (hereafter referred to as “Hybrid-B”) (Fig. 2b), both of which have been reported in a yeast model^39^. If these Hybrid complexes form in the hypothesized combinations, it is expected that they can simultaneously interact both with Rab5 and Rab2 or Rab7/Arl8 and Rab5 at each end to exert heterotypic tethering of vesicles, which are distinctive functions from HOPS and CORVET.

First, we tested the formations of the Hybrid complexes by exogenously expressing the subunits of each combination. Since it has been reported that a mutation in VPS16 affects HOPS and CORVET stability^40^, we used cells stably expressing V5-VPS16. To demonstrate the existence of Hybrid-A or Hybrid-B, we transiently expressed the VPS family proteins with different tags (Myc-VPS41/Myc-VPS8, His-VPS18, FLAG-VPS11, HA-VPS39/HA-VPS3) in the expected combinations (Fig. 2c). After immunoprecipitation (IP) of Myc-tagged VPS41 or VPS8, the inclusion of each subunit in the IP sample was checked by the anti-tag antibodies.

By IP with Myc-VPS41, class C core and VPS39 (composing HOPS), and class C core and VPS3 (composing Hybrid-A) were detected (Fig. 2d). Similarly, class C core and VPS3 (composing CORVET), and class C core and VPS39 (composing Hybrid-B) were immunoprecipitated by Myc-VPS8 (Fig. 2e). These results indicate that in addition to HOPS and CORVET, both of the two Hybrid complexes we expected could be formed in cells if each combination of the required subunits were expressed.

**Figure 3:**
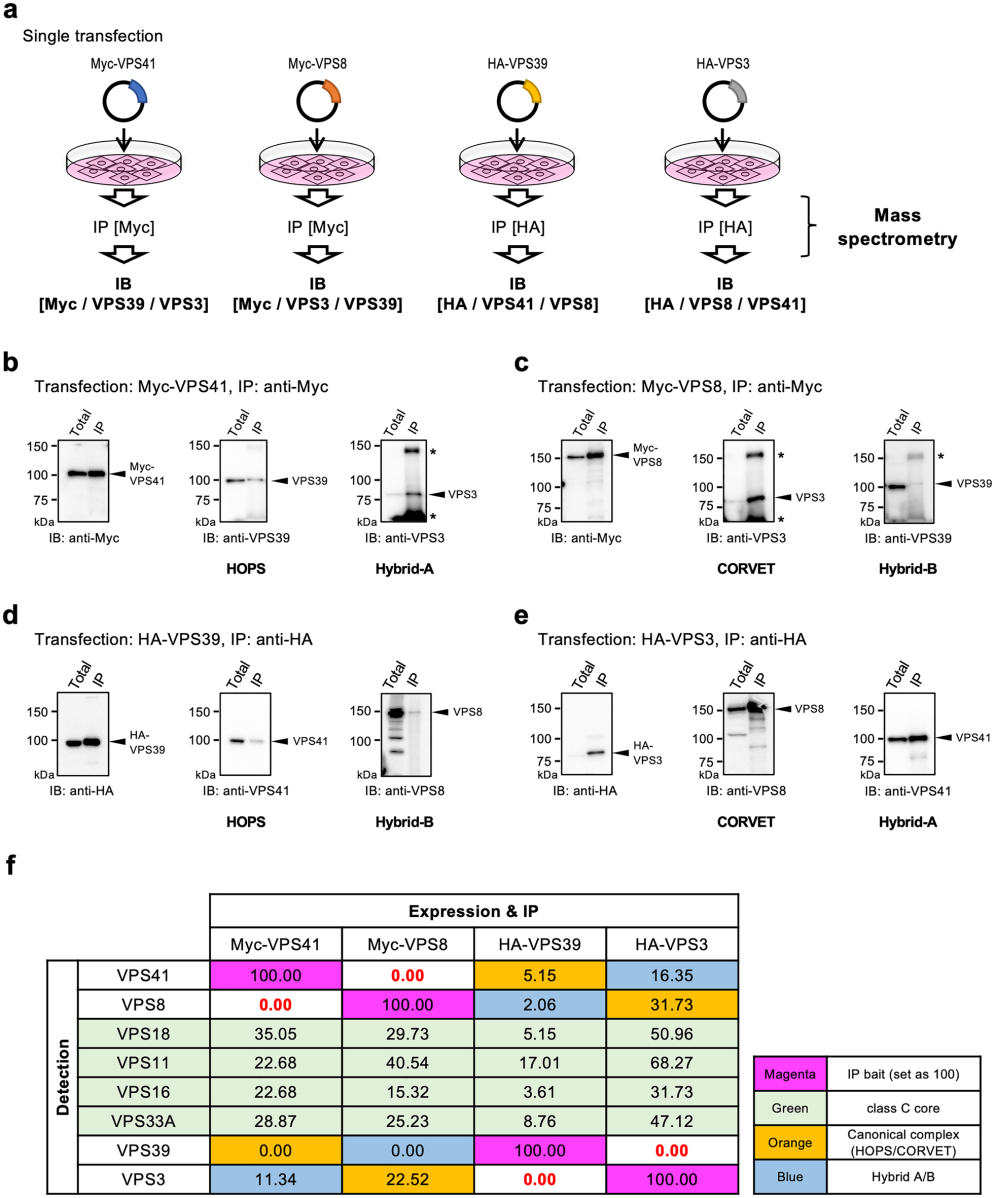
Detections of the endogenous canonical and non-canonical tethering complexes by mass spectrometry. (**a**) An experimental workflow. One tagged-VPS protein at either end of the tethering complex was over-expressed in WT HeLa-Kyoto cells. Endogenous proteins interacting with exogenously expressed VPS41, VPS8, VPS39, or VPS3 were pulled down by anti-Tag antibody and Protein-G magnetic beads. (**b**–**e**) IP-check before Mass spectropetry analysis by western blotting. Interactions with the expected partner proteins for VPS41 (**b**), VPS8 (**c**), VPS39 (**d**), and VPS3 (**e**) were confirmed by immunoblotting (arrowheads). The relevant complex is shown at the bottom for each panel. Asterisks (*) represent non-specific bands. (**f**) The relative abundance of each tethering complex subunit based on the signal intensities from the mass spectrometric analysis is shown. Each value is shown as a relative value to the over-expressed subunit which served as the bait as 100. The meaning of the background colouring of cells is shown in the legend on the right side.

Molecular interactions resulting from protein overexpression are artificial experimental systems and may not always reflect physiological protein–protein interactions. The tag-IP results shown in Fig. 2 encouraged us to prove the existence of the Hybrid complexes by further analysis with mass spectrometry. We tested whether IP-western blotting could detect endogenous HOPS, CORVET, Hybrid-A, or Hybrid-B complexes in WT HeLa cells by single expression of VPS41, VPS39, VPS8, or VPS3 (Fig. 3a). Endogenous VPS39 and VPS3 were co-immunoprecipitated when Myc-VPS41 was expressed and immunoprecipitated, which suggests the existence both of HOPS and Hybrid-A (Fig. 3b). Similarly, when Myc-VPS8 was expressed and immunoprecipitated, endogenous VPS3 and VPS39 were co-immunoprecipitated, which indicates the presence of CORVET and Hybrid-B, although the amount of Hybrid-B was low (Fig. 3c). To further confirm these endogenous tethering complex formations and exclude the artefacts by the expression of the “bait” protein, immunoprecipitation with VPS39 or VPS3, which is located on the opposite end was also tested. When HA-VPS39 was expressed and immunoprecipitated, endogenous VPS41 and VPS8 were detected, respectively (Fig. 3d). This indicates the presence of HOPS and Hybrid-B. Again, similarly, when HA-VPS3 was expressed and immunoprecipitated, endogenous VPS8 and VPS41 were detected, indicating the presence of CORVET and Hybrid-A (Fig. 3e). These results suggest that endogenous levels of VPS family proteins seem to be enough to form Hybrid-A and Hybrid-B complexes.

Next, we performed mass spectrometry analysis of the immunoprecipitated samples to verify whether complexes such as Hybrid-A and Hybrid-B are formed (Fig. 3a). In samples immunoprecipitated with Myc-VPS41 or Myc-VPS8, endogenous VPS3 was detected along with the class C core, suggesting the presence of Hybrid-A and CORVET (Fig. 3f). In addition, VPS41 and VPS8 were detected both in the immunoprecipitated samples with HA-VPS39 and HA-VPS3, supporting the intrinsic presence of all four complexes including Hybrid-A and Hybrid-B (Fig. 3f). Notably, VPS41 and VPS8, or VPS39 and VPS3 were detected exclusively, indicating that HOPS and Hybrid-A or CORVET and Hybrid-B exist as independent complexes (Fig. 3f, shown in red number). From the results of mass spectrometry, VPS39 was not detected in the VPS8 IP sample. In order to rule out the possibility that Hybrid-B, whose abundance was considered to be low, would not have formed without the overexpression of constitutive subunit(s), we also examined whether a completely endogenous Hybrid-B was present. We immunoprecipitated VPS8 from WT cell extracts and VPS39 was detected by western blotting (Supplementary Fig. 3). Based on these results above, we concluded that the hybrid complexes are indeed formed in the cells in addition to canonical tethering complexes (Fig. 3b–f).

### The Hybrid complexes function in pinocytosis

To test whether the Hybrid complexes have specific functions, we attempted to generate double KO (dKO) cells lacking two of the Rab-binding VPS genes, as the single KO leaves two of the four complexes in cells and thus makes it difficult to fully uncover the function of the novel Hybrid complexes. We could obtain VPS41+39 dKO cells (Only CORVET is presumed to remain out of the four complexes), VPS41+3 dKO cells (only Hybrid-B remains), and VPS8+3 dKO cells (only HOPS remains); however, we could not get VPS8+39 dKO cell (only Hybrid-A remains) after several trials (Supplementary Fig. 4).

**Figure 4:**
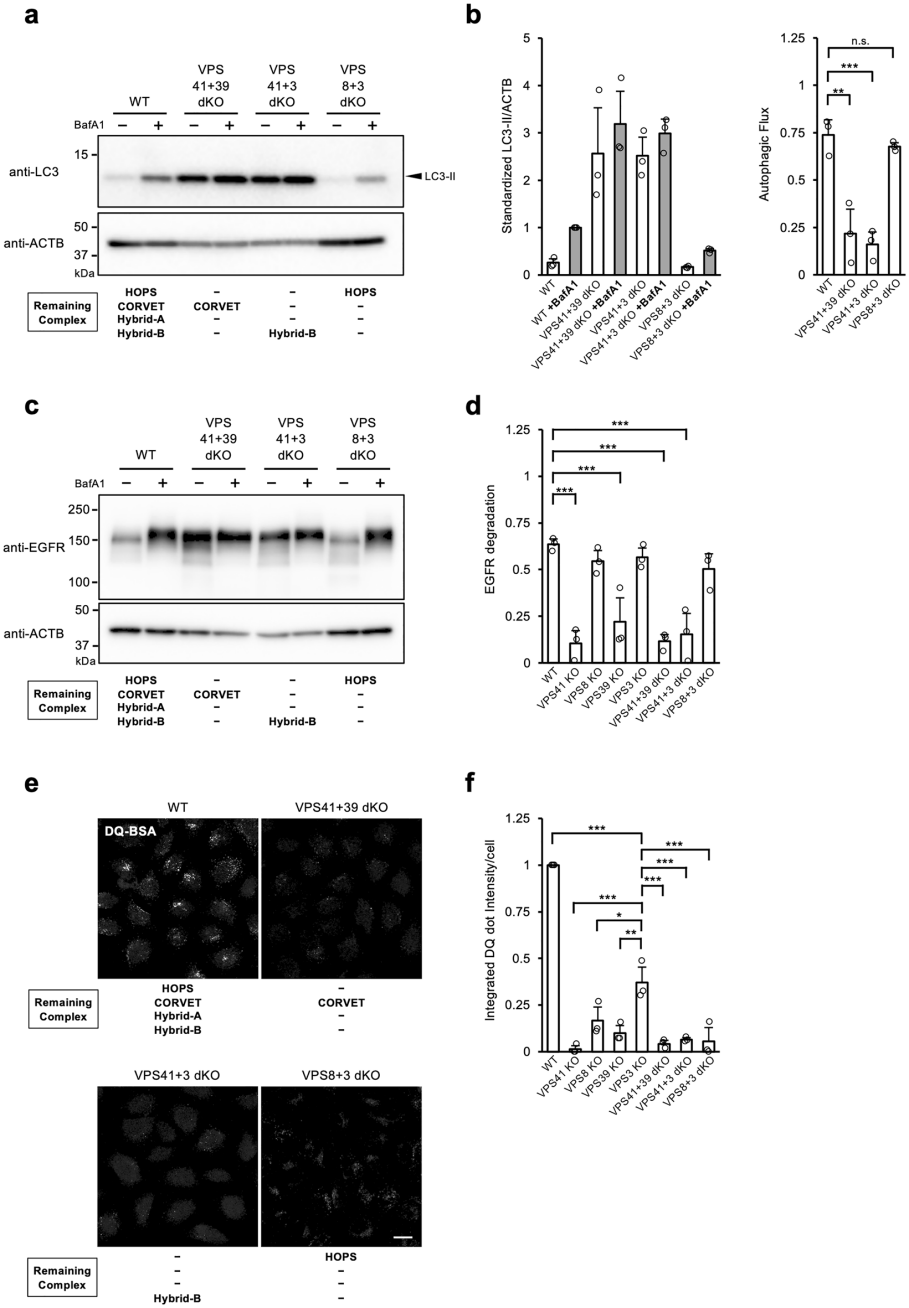
Involvement of the Hybrid complexes in endocytosis progression. (**a**) Autophagic flux in the VPS dKO cells. Cells were cultured in the absence or presence of BafA1 for 2 h. The whole cell lysates were analysed by western blotting. The theoretical remaining complexes for each cell line are shown at the bottom. A representative result out of independent 3 experiments is shown. (**b**) A quantitative analysis of the immunoblot is shown in panel (**a**). The mean and S.D. of intensities and autophagic flux from 3 independent results are shown in the left panel and right panel, respectively. (**c**) EGFR endocytosis in VPS dKO cells. WT and VPS KO cells were stimulated with 100 ng/mL of EGF for 2 h in the absence or presence of BafA1. The whole cell lysates were analysed by western blotting. A representative blot is shown from 3 independent experiments. (**d**) Quantitative analysis of band intensities in panel (**c**). Mean and S.D. from 3 independent experiments are shown. The overlayed circle represents each data point. Statistics were calculated by one-way ANOVA followed by post hoc Tukey’s test. (**e**,**f)** DQ-BSA uptake. VPS dKO cell lines were treated with DQ-BSA containing medium for 4 h. A representative image of DQ-BSA for each cell line is shown (**e**). Quantitative data of relative DQ dot intensity/cell is displayed. The data is shown as an average of the median from three independent experiments (**f**). Statistics were calculated by one-way ANOVA followed by post hoc Tukey’s test. *Scale Bar* = 10 µm. *p < 0.05, **p < 0.01, ***p < 0.001, and n.s.; not significant.

Using these dKO cell lines, we tested autophagic flux analysis to examine the function of each complex in autophagy. We found that VPS8 + 3 dKO showed autophagic flux levels comparable to that of WT cells. On the other hand, autophagic flux was significantly impaired in VPS41+39 dKO cells and in VPS41+3 dKO cells (Fig. 4a,b, Supplementary Fig. 4). These results indicate that only HOPS functions as a tethering complex in autophagic degradation and that CORVET and the Hybrid complexes are not required for autophagy.

We next investigated receptor-mediated endocytosis with these dKO cells. We found that the endocytic degradation of EGFR in VPS41+39 dKO cells and VPS41+3 dKO cells were significantly reduced compared with WT, while VPS8+3 dKO cells did not show a difference from WT cells (Fig. 4c,d). These results suggest that HOPS is enough to drive EGFR endocytosis. VPS41 single KO cells lacking HOPS had shown much-reduced endocytosis (Fig. 1c,d); thus, it would make sense that VPS41+39 dKO cells and VPS41+3 dKO cells could not complete EGFR endocytosis in the absence of HOPS.

We further investigated pinocytosis activities in VPS dKO cells using DQ-BSA and found that pinocytosis was almost stopped in all dKO cells tested (Fig. 4e,f). VPS3 KO cells showed reduced, but maintained pinocytosis activity, while VPS8 KO, VPS8+3 dKO, VPS41 KO, VPS41+3 dKO, and VPS39 KO cells largely lost it at comparable levels. This means that pinocytic degradation is nearly stopped by the loss of either HOPS or Hybrid-B (or lack of both), but can be maintained in the presence of both HOPS and Hybrid-B complexes. Additionally, the reduced level of pinocytosis observed in the VPS3 KO cells suggests the contribution of CORVET and/or Hybrid-A complexes in facilitating this pathway, albeit we could not highlight the stand-alone function of Hybrid-A. Nevertheless, it is noteworthy that these observations in pinocytosis completely differed from that of receptor-mediated endocytosis.

### Endocytosed EGF and dextran differently localise in HOPS-deficient cells

The present study showed the contributions of Hybrid-B in pinocytosis, while it has minimal impacts on receptor-mediated endocytosis. This suggests these two types of endocytosis use different vesicular trafficking mechanisms after substrate uptake. Therefore, the behaviour of the transport vesicles containing each cargo during endocytosis may also differ from each other. To test this, we simultaneously incorporated fluorescence-labelled EGF as a substrate for receptor-mediated endocytosis and fluorescence-labelled dextran as a substrate for pinocytosis into WT cells and then observed the time course of their transport into lysosomes. The results showed that the colocalisation of the two increased rapidly 15 min after substrate addition, and then reached a plateau state after 60 min. The colocalisation of each substrate with LAMP1-positive vesicles showed a distinct time course in the beginning, but both gradually increased after 30 min. These results suggest that both EGF and dextran reach lysosomes after approximately 60 min (Supplementary Fig. 5).

**Figure 5:**
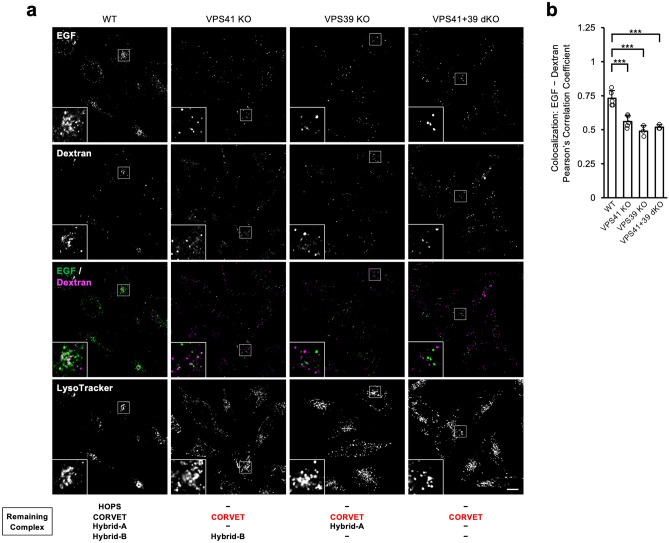
Different localisation of receptor-mediated endocytosis and pinocytosis cargo. (**a**) Dual tracing of trafficking processes of receptor-mediated endocytosis and pinocytosis substrates in VPS KO cells. The cells were treated with Alexa Fluor 488-labelled EGF and Alexa Fluor 647-labelled dextran-containing medium for 1 h. Fluorescence images derived from the incorporated substrates were captured using a confocal microscope. Representative images are shown. *Scale bar* = 10 µm. (**b**) Quantitative analysis of colocalisation between endocytosed EGF and dextran in (**a**). At least, five images for each cell line were captured and analysed. Pearson’s correlation coefficient of each cell was plotted. ***p < 0.001.

Having demonstrated that the two endocytic substrates were sufficient to reach the lysosomes in approximately 60 min in WT cells, we next used VPS41, VPS39 KO, and VSP41+39 dKO cells, which lack HOPS and endosome-lysosome fusion and treated them with EGF and dextran to trace their fate until 60 min^41^. EGF and dextran represented similar localisation and both of them also colocalised with LysoTracker™ signals in WT cells at 60 min as previously observed. On the other hand, these two substrates showed different localisations in VPS41 KO, VPS39 KO, and VPS41+39 dKO cells in separated locations from lysosomal compartments at the 60 min mark (Fig. 5a,b). These results suggest that the pathways for transferring substrates of receptor-mediated endocytosis and pinocytosis are different from each other before reaching lysosomes.

### Hybrid-B functions in the vesicle trafficking of pinocytosed cargo

Our study has suggested that at least Hybrid-B is required for the degradation of substrates taken up by pinocytosis in addition to HOPS. To investigate the contribution of the Hybrid complexes to the kinetics of pinocytosis, we observed the progression of dextran transport to lysosomes.

**Figure 6:**
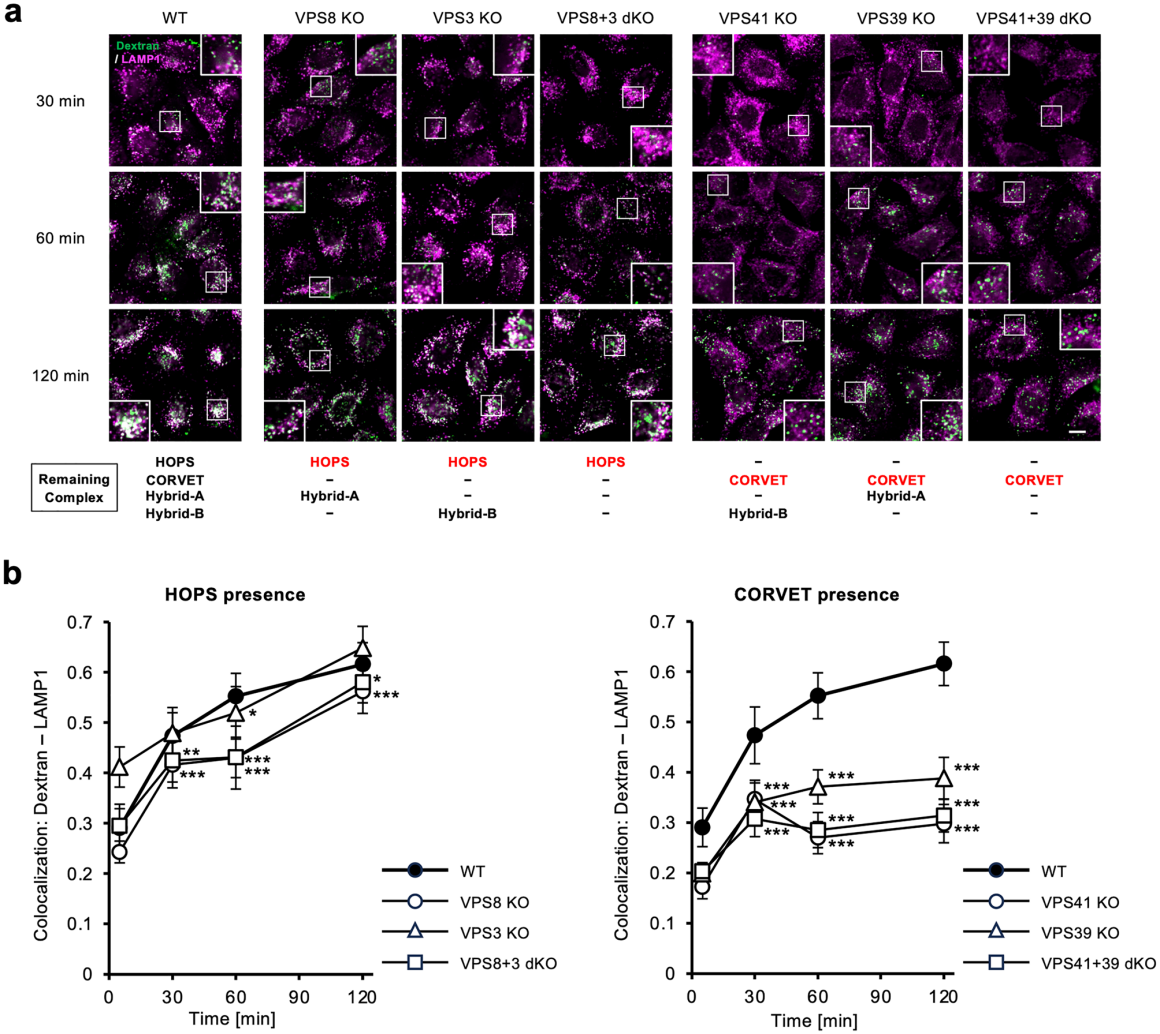
Time course analysis of pinocytic cargos in the VPS KO and dKO cells. (**a**) Tracing of trafficking processes of pinocytosis substrates in VPS KO and dKO cells. The cells were treated with Alexa Fluor 647-labelled dextran-containing medium for 15 min, 30 min, or 60 min, then fixed and stained for LAMP1. Fluorescence images derived from the dextran (green) and LAMP1 (magenta) were captured using a confocal microscope. Representative images are shown. *Scale bar* = 10 µm. (**b**) Quantitative analysis of colocalisation between the endocytosed dextran and LAMP1 in (**a**). Five images were obtained for each cell line to obtain approximately 50 cells to measure. Pearson’s correlation coefficient was determined cell by cell, and the median ± S.D. for each cell line and each time point was plotted on time series line graphs. Statistical analyses were done by the Kruskal–Wallis test followed by the Mann–Whitney U test with Holm correction. Significance against WT for each time point is shown as; *p < *0.05*, **p < 0.01, ***p < 0.001.

We examined the time course of colocalisation of the fluorescence-labelled dextran with LAMP1-positive vesicles at 30, 60 and 120 min. The colocalisation coefficient in VPS3 KO (HOPS and Hybrid-B remain) cells was comparable to WT, whereas those in VPS8 KO (HOPS and Hybrid-A remain) and VPS8+3 dKO (HOPS only) cells were significantly lower than WT (Fig. 6a,b). This is consistent with the results of the DQ-BSA experiments which reflect lysosomal degradation (Fig. 4e,f). On the other hand, in VPS41 KO, VPS39 KO, and VPS41+39 dKO cells which lack HOPS, the colocalisation coefficients remained low until 120 min, suggesting dextran was not transported to LAMP1-positive vesicles. This also matches the data that DQ-BSA degradation does not proceed under the conditions of HOPS absence (Fig. 4e,f).

To summarize our present study, the two Hybrid complexes—which consist of the Class C core and different combinations of subunits at both ends from HOPS or CORVET—were identified. Furthermore, it was found that the pathways to lysosomes in receptor-mediated endocytosis and pinocytosis are independent and that the newly identified Hybrid-B functions in the pinocytic pathway.

## Discussion

The tethering complexes involved in autophagy and endocytosis pathways have been extensively studied^5,7,36^. However, this is the first time that the "Hybrid" complexes, Hybrid-A and Hybrid-B, where components of HOPS or CORVET are interchanged, have been shown to exist in mammalian cells.

Since VPS41 and VPS8 share the binding counterparts (VPS33A + VPS16 + VPS18) as shown in Fig. 2a, it was anticipated that a complex swapping its subunit between VPS41 and VPS8 may exist. This can also be applied to VPS39 and VPS3, binding with VPS11. Therefore, provided all the necessary subunits are expressed in the cell, it is possible that complexes such as Hybrid-A and Hybrid-B can be formed. On the other hand, Peplowska et al*.* have already reported that such hybrid-type complexes can be formed as intermediates in the equilibration between HOPS and CORVET transitions in yeast^39^, and Lőrincz et al. claimed that overexpression of VPS8 dislodges VPS41 from HOPS^42^. Their reports suggest another possibility that the Hybrid complexes are formed by the equilibrium transitions among the complexes. In either model, the formation process of the Hybrid complex can be explained and is consistent with our results. Mass spectrometry results showed that VPS8 was not detected in the VPS41 IP sample, while VPS41 was not detected in the VPS8 IP sample. Similarly, VPS3 was not detected when IP with anti-VPS39 and VPS39 was not detected when IP with anti-VPS3 (Fig. 3f). These results suggest that VPS41 and VPS8, or VPS39 and VPS3 are competing with each other, and are exclusively bound to the class C core, respectively. This means that the four complexes; HOPS, CORVET, Hybrid-A and Hybrid-B exist independently.

In addition to confirming the physical feasibility of Hybrid complex formation by IP-western blotting experiments (Fig. 2) in an overexpression system, as in previous studies^39,42^, in the present study, we also attempted to detect the endogenous subunits of the tethering complexes by mass spectrometry (Fig. 3f). Since the Myc- or HA-tagged bait proteins were overexpressed for immunoprecipitation of the respective target complex, it is not proper to say that we detected the strictly endogenous complex even in this experiment. Nevertheless, considering that CORVET and Hybrid-A were detected in immunoprecipitation from both ends (with VPS8 or VPS3 and with VPS41 or VPS3), we conclude that these endogenous complexes do exist. VPS39 was not detected by mass spectrometry from the IP sample with Myc-VPS41 or with Myc-VPS8 (Fig. 3f); however, VPS-41 (HOPS) or VPS8 (Hybrid-B) was detected in the IP sample with HA-VPS39 in mass spectrometry, so the existence of HOPS and Hybrid-B is not necessarily denied. We have applied a high probability threshold in the analysis of peptides by mass spectrometry in order to obtain accurate results. Under this condition, the amounts of proteins may have been underestimated. Therefore, it is suggested that the discrepancy between the results of IP-western blotting and mass spectrometry is due to differences in the protein amounts and detection sensitivities of the different experiments^36^.

Peplowska et al. previously described the existence of hybrid-type complexes in yeast and showed that overexpression of VPS3 converts HOPS into “i-CORVET” (corresponding to Hybrid-A in this manuscript), and then “i-CORVET” into CORVET upon overexpression of VPS8^39^. They proposed a model in which there is a directional equilibrium between CORVET and HOPS via an intermediate “i-CORVET” or “i-HOPS” (corresponding to Hybrid-B). As the number of complexes may be affected by overexpression of subunits, we believe that data obtained in overexpression systems should be interpreted with caution and that further studies are needed on the transition between canonical and Hybrid complexes. In the present study, the presence of fully endogenous Hybrid-B was also confirmed (Supplementary Fig. 3). Based on these results and considerations, we concluded that the Hybrid complexes are actually formed in the cells as well as canonical tethering complexes.

To check whether the new complexes we identified are more than just an intermediate in the transition between HOPS and CORVET but have physiological functions, we examined the contribution of the hybrid complexes to endocytosis. Our results indicated that the absence of Hybrid complexes does not affect receptor-mediated endocytosis, but does affect pinocytosis. Comparing the cells deficient in VPS family molecules generated by the CRISPR/Cas9 system, pinocytosis was significantly impaired in VPS8+3 dKO cells (HOPS only remains) and VPS41+3 dKO cells (Hybrid-B only remains). On the other hand, pinocytosis was maintained at ~ 40% of WT in VPS3 KO cells (HOPS and Hybrid-B remain) (Fig. 4e–f). This means Hybrid-B has a distinct role in pinocytosis from HOPS. The ~ 60% reduction in DQ-BSA degradation in VPS3 KO cells compared to WT cells is presumably due to the function of depleted CORVET and Hybrid-A. However, it is not possible to determine which contribution is responsible for the reduction in the present study.

Since it is impossible to generate cells in which only the combination of HOPS and CORVET is present in our experimental system, we cannot discuss whether the Hybrid complexes are substitutes for CORVET or have a unique function. Despite the fact that Hybrid-B is far less abundant than CORVET (Fig. 3f), the effect of Hybrid-B in pinocytosis was apparent, which may suggest the high functionality of Hybrid-B in pinocytosis. If Hybrid-B interacts with Rab5 and Rab7 in VPS8 and VPS39 ends, respectively, Hybrid-B may function in facilitating heterotypic fusion between Rab5 and Rab7 vesicles in the pinocytic pathway; HOPS is known as functioning in homotypic fusion between Rab7 vesicles^41^. Hybrid-A was shown to be abundant; however, we were unable to demonstrate a clear functional role of Hybrid-A in the present study.

We tried multiple times but could not obtain a single clone of VPS8 and VPS 39 dKO cells (cells thought to have only Hybrid-A). Similarly, we could not establish VPS18 KO and VPS11 KO cells, which are common to all complexes. This could be because the cells cannot survive if the complexes were not present at all or only Hybrid-A was present. This implies that Hybrid-A does not have the ability to maintain critical cellular functions. We hope that as studies follow up from the perspective of Hybrid-A and B in the future, the Hybrid-specific functions will become more apparent.

With respect to autophagy, its activity was not influenced by the presence or absence of CORVET and Hybrid complexes and was explained by the presence of HOPS (Figs. 1a,b and 4a,b). These results might not exclude the possibility of the Hybrids' involvement in autophagy, but we could not find out any impact of the Hybrid complexes on autophagy progression under this experiment setting.

In the previous models for vesicular transport, receptor-mediated endocytosis and pinocytosis cargoes are loaded into distinct vesicles at the time of uptake, but subsequently merge and are transported to lysosomes. However, our finding that the involvement of canonical tethering complexes and Hybrid complexes in lysosomal degradation differs between receptor-mediated endocytosis and pinocytosis suggests that these pathways of vesicular transport in endocytosis do not merge until later in the process. To confirm this, we traced two different cargoes simultaneously. Time-course observations in WT cells showed that the colocalisation of EGF and dextran increased with time and almost reached a plateau at 60 min, when they also colocalised with LAMP1, suggesting that both cargoes reached the lysosomes at 60 min. HOPS is known to mediate tethering between late endosomes and lysosomes. Therefore, we checked whether the cargo of receptor-mediated endocytosis merged with the cargo of pinocytosis by tracking the cargo for 60 min in VPS41KO, VPS39 KO and VPS41+39 dKO cells, where HOPS was absent. The results showed that EGF and dextran did not colocalise (Fig. 5). These results support our idea that receptor-mediated endocytosis and pinocytosis endocytic pathways are independent until just before these vesicles fuse with lysosomes. This interesting fact has not been well characterized in mammalian cells so far. In the present study, we focused not on the endosomal markers, but on the distinct cargos internalisation through different modes and tracked them in the same cell. In addition, we have generated a variety of KO cells with genome editing technology and observed them in the same assay system, which aided us in realizing our findings. The identification of marker proteins that define these two pathways will expand research, and their significance will become clear in the future.

We attempted to further clarify the role of the Hybrid complexes in pinocytosis and observed cargo trafficking in the KO cells with time course. Observations of VPS KO cells in the series with residual HOPS (Fig. 6) showed that dextran accumulated in lysosomes in VPS3 KO cells at a level comparable with those of WT cells; however, the accumulations were reduced in VPS8 KO cells and in VPS8+3 dKO cells. This suggests that the Hybrid-B facilitates the progression of pinocytosis. On the other hand, the accumulation of dextran in lysosomes did not progress in cells of the HOPS-deficient series (Fig. 6). Taken together, these results suggest that the contribution of Hybrid-B as well as HOPS play an important role in pinocytosis, and are consistent with the results of DQ-BSA (Fig. 4e,f). Strictly speaking, dextran transport to lysosomes in VPS8+3dKO and VPS8 KO cells, where HOPS is present, appears to behave differently from that in VPS41 KO, VPS39 KO and VPS41+39 dKO cells, where HOPS is absent. Whilst lysosomal transport completely stops in the absence of HOPS, Hybrid-B is not essential in the pinocytosis pathway as transport also takes place gradually in the absence of Hybrid-B (in the presence of HOPS). These results suggest that Hybrid-B works to increase the efficiency of pinocytosis. On the other hand, this leakage of transport was not captured as statistically significant in the DQ-BSA degradation experiments, most likely due to the sensitivity of the DQ fluorophores.

From these results described above, it seems certain that different vesicle tethering systems are used in different endocytic pathways, and it supports the physiological functions of the Hybrid complexes we have identified in this study. In addition to HOPS and CORVET, several other tethering complexes such as CHEVI, FERARI, and mini-CORVET have been reported to be involved in vesicular trafficking networks including endocytic, recycling, Golgi, or retrograde transportation pathways^7,43–45^. Their interactions or involvement with as of yet unknown tethering complexes might achieve the precise regulation of vesicular trafficking in the cell. Further functional and molecular characterization of these tethering molecules, including the Hybrid complexes of this study, is necessary to elucidate the full picture of vesicle trafficking regulations by tethering complexes and to understand diseases caused by their abnormalities.

## Methods

### Cell culture

HeLa Kyoto cells (simply described as HeLa cells in this manuscript) were cultured in Dulbecco’s Modified Eagle Medium (DMEM) (Sigma-Aldrich, #D5796) supplemented with 10% heat-inactivated fetal bovine serum (FBS) and 100 units/mL penicillin—100 µg/mL streptomycin (Nacalai-Tesque, #26253-84) in a 5% humidified CO_2_ incubator at 37 °C.

### Plasmids

VPS41, VPS8, VPS39, VPS3, VPS18, and VPS11 cDNA were amplified from HeLa Kyoto cDNA by PCR and then cloned into the pcDNA3.1 expression vector (Thermo Fisher) with the tag peptide sequence. The insert sequences were confirmed by DNA sequencing.

### Stable gene expression with viral transduction

VPS16 cDNA was amplified from HeLa Kyoto cDNA by PCR. After tagging with the V5 sequence, the cDNA was cloned in pMXs-IRES-puro retroviral vector. Plat-E cells were transiently transfected with the V5-VPS16 retrovirus vector with pCG-VSV-G using PEI reagent. After 48 h-culturing, the culture supernatant was collected and cleared with a 0.22 µm syringe filter. HeLa-Kyoto cells were infected with the recombinant virus in the presence of 10 µg/mL of polybrene for 24 h. The infected cells were selected with 1 µg/mL of puromycin for 3 passages. The expression of exogenous VPS16 was confirmed by immunoblotting.

### Genome editing by CRISPR/Cas9

For preparing KO cells the guide sequences were designed using Benchling CRISPR Guide RNA (gRNA) Design Tool (https://www.benchling.com/crispr/) or CRISPR direct^46^ (https://crispr.dbcls.jp/). The list of guide sequences was shown in Supplementary Table 2. Double-stranded DNA coding guide RNA sequences were prepared by annealing synthesized complementary DNA oligos on a thermal cycler, and then cloned into the pSpCas9(BB)-2A-GFP (pX458) vector (Addgene plasmid #48138). The targeting plasmid was transfected into HeLa Kyoto cells with Effectene Transfection reagent (Qiagen, #301425) or with PEI Max (Polysciences, #24765). After 48 h transfection, GFP^+^ populations were sorted into single cell cultures by FACSAria III Cell Sorter (BD Biosciences). The cell clones were left to grow in the 96-well plates (Corning) for 2 weeks. Genomic DNA from each clone was purified with a QuickExtract™ DNA Extraction Solution (Lucigen), and then the genome sequence of the targeted site was analysed by Sanger sequencing (Supplementary Table 2). The deletion of the target protein expression was confirmed by western blotting (Supplementary Figs. 1 and 4).

### Antibodies

Primary antibodies used for immunoblotting: anti-ACTB (MBL, #M177-3, 1:3000), anti-EGFR (MBL, #MI-12-1, 1:3000), anti-LC3 (MBL, #PM036, 1:3000), anti-VPS41 (SCBT, #sc-377118, 1:1000), anti-VPS8 (Proteintech, 15079-1-AP, 1:2000), anti-VPS39 (SCBT, sc-514762, 1:1000), anti-VPS3 (TRAP-1) (SCBT, #sc-13134, 1:1000), anti-VPS18 (Proteintech, #10901-1-AP, 1:2000), anti-VPS11 (SCBT, sc-515094, 1:2000), anti-VPS16 (Proteintech, # 17776-1-AP, 1:2000), anti-VPS33A (NovusBio, #NBP2-20872, 1:1000), anti-FLAG (Sigma-Aldrich, #F1804, 1:1000), anti-HA (Gene Tex, #GTX18181, 1:2000), anti-His (MBL, #PM032, 1:1000), anti-Myc (MBL, #M192-3, 1:10000), anti-V5 (MBL, #M215-3, 1:2000), anti-FLAG HRP-conjugated (FUJIFILM-Wako, # 019-22394, 1:10000), anti-Myc HRP-conjugated (MBL, #M192-7, 1:10000), anti-HA HRP-conjugated (MBL, #M180-7, 1:10000). Secondary antibodies used for immunoblotting: HRP-conjugated anti-rabbit IgG (Invitrogen, #31460, 1:3000 or 1:10000) and anti-mouse IgG (Invitrogen, #31430, 1:3000 or 1:10000).

Primary antibodies used for immunofluorescence staining: anti-LC3 (MBL, #PM036, 1:500. Secondary antibodies used for immunofluorescence staining: Alexa Fluor 488 anti-rabbit IgG (H + L) (Molecular Probes, #A21206).

### Immunoblotting

Cells were directly lysed in sodium dodecyl sulfate (1 × SDS) sample buffer (250 mM Tris–HCl (pH 6.8), 40% (w/v) glycerol, 6.2% (w/v) dithiothreitol, 8% (w/v SDS), bromophenol blue), heated at 95 °C for 5 min, followed by shearing gDNA on a tube shaker at room temperature for 5 min. The treated samples were separated by SDS-PAGE and transferred onto polyvinylidene fluoride (PVDF) membranes (Merck Millipore). The membranes were blocked with 1% skimmed milk in TBS-T (Tris-buffered saline with 0.5% Tween 20) for 1 h, followed by incubating with primary antibodies for 1 h at room temperature or 4 °C overnight. After serial washings with TBS-T, the membrane was incubated with HRP-conjugated secondary antibody for 1 h at room temperature and again washed 3 times. Immobilon Forte (Merck Millipore, # WBLUF0500) was used as a substrate to have chemiluminescence and obtained the images of immunoreactive bands with ChemiDoc Touch (Bio-Rad) and quantified with Image Lab (Ver. 6.1.0, Bio-Rad). Some membranes were cut into two or more pieces by molecular weight to incubate with different antibodies in parallel. Full-size blot images are shown in Supplementary Information.

### Immunofluorescence analysis

Cells were grown on coverslips, washed with PBS, and fixed with 4% paraformaldehyde (PFA) for 15 min. After fixation, cells were permeabilized by 50 μg/mL digitonin for 15 min. The coverslips were first incubated for 1 h with primary antibodies, washed three times with PBS, and then incubated for 1 h with appropriate fluorochrome-labelled secondary antibodies. DAPI (DOJINDO, #D523) and HCS Cell Mask Deep Red (Thermo Fisher, #H32721) were included in the secondary antibody for LC3 dot flux analysis. All steps were conducted at room temperature. The fluorescent images were captured under LSM700 confocal microscope (Carl Zeiss). Image analysis was performed using the ImageJ software (National Institutes of Health, USA) or CellProfiler™^47^ (https://cellprofiler.org/releases).

### Autophagic flux assay

Autophagic flux assay was performed as described previously^48^. Briefly, cells were seeded and cultured one day before the assay. The confluency of cells was adjusted approximately to 70% at seeding to achieve confluent condition on the day of assay. The medium was replaced with pre-warmed flesh one without 125 nM Bafilomycin A1 (BafA1, Cayman Chemicals, #11038). After 2 h incubation, cells were washed with PBS twice and lysed in SDS sample buffer. The samples were analysed by immunoblotting using anti-LC3 (MBL, #PM036) and anti-ACTB (MBL, #M177-3) antibodies.

### EGFR degradation assay

Cells were washed twice with pre-warmed DMEM and incubated in an FBS-free medium for 2 h before stimulation. Additionally, cells were treated with or without 125 nM of BafA1 for 1 h before stimulation. Cells were stimulated with 100 ng/mL of EGF (Thermo Fisher, #53003-018) for 2 h in the presence or absence of BafA1. After 2 h of incubation, cells were washed with PBS and lysed in an SDS sample buffer. Samples were subsequently analysed for EGFR and ACTB by immunoblotting.

### Endocytosis tracking

To trace pinocytosis substrates, cells were cultured in the presence or absence of either 200 µg/mL of DQ Red-BSA (Thermo Fisher #D12051), or 50 µg/mL of Alexa Fluor 488-BSA (Thermo Fisher, #A13100), or 100 µg/mL of Alexa Fluor 647-dextran 10 kDa (Thermo Fisher, #D22914) for the indicated time. As for receptor-mediated endocytosis, cells were pre-cultured in serum-free DMEM for 2 h before loading substrates. After serum starvation, the cells were loaded with 1 µg/mL of Alexa Fluor 488-EGF and 100 µg/mL of Alexa Fluor 647-dextran simultaneously and then incubated in a CO_2_ incubator at 37 °C for 1 h. After cultivation, the cells were washed with PBS twice and then fixed with 4% PFA. The images were observed and captured under a confocal microscope LSM700.

### Immunoprecipitation

Cells were transiently transfected with the VPS gene expression plasmids with PEI max. The total amount of plasmid DNA introduced was adjusted to the same using pcDNA3.1 empty vector (Thermo Fisher). After 48 h, cells were washed twice with cold PBS and then resuspended with 1 mL of PBS supplemented with a protease inhibitor cocktail (Roche, #11873580001). Cells were homogenized by passing a 27G needle (TERUMO) 20 times on ice and centrifuged at 15,000 rpm for 10 min at 4 °C. The protein concentration of the cleared lysate was determined by Bradford assay (QuickStart Dye reagent, Bio-Rad, #500-0205). Equal amounts of proteins among samples were used for the immunoprecipitation experiment. The lysate was incubated with anti-Myc (MBL, #M192-3), anti-HA (GeneTex, #GTX18181), or anti-VPS8 (Proteintech, #15079-1-AP) antibody on ice for 1 h and then cleared by centrifugation again. Protein G magnetic beads (Cytiva, #28951379) were added and incubated at 4 °C with rotation overnight. The beads were washed with cold PBS 4 times, and bound proteins were eluted with 2 × SDS sample buffer with heating at 95 °C for 5 min. Resultant eluates were analysed either by immunoblotting or mass spectrometry.

### Mass spectrometry

Mass spectrometry was performed using a commissioned analysis service provided by MS Bioworks (Ann Arbor, MI). Briefly, Myc- or HA-tagged proteins indicated were transiently expressed in HeLa cells, and immunoprecipitated with anti-Myc or anti-HA antibodies and magnetic beads. Proteins on the beads were eluted using 2 × sample buffer and submitted to MS Bioworks in freezing conditions. Samples were resolved in SDS-PAGE and then digested in the gel by trypsin before LC–MS/MS. Protein identification was performed by MASCOT software (Matrix Science) and analysed by Scaffold 5 (Proteome Software). Contaminants such as keratin, immunoglobulins, and trypsin were excluded from the analysis.

### Statistics and reproducibility

The one-way ANOVA followed by a post hoc Tukey test for parametric analyses and the Kruskal–Wallis test followed by the Mann–Whitney U test with Holm adjustment for non-parametric analyses were performed with EZR^49^, a modified version of R commander designed to add statistical functions frequently used in biostatistics (The R Foundation for Statistical Computing, Vienna, Austria). Sample sizes for each analysis are indicated in the Figure legends. For all tests, a *p*-value of less than 0.05 was considered statistically significant. (*: *p* < 0.05; **: *p* < 0.01, ***: *p* < 0.001).

### Supplementary Information

Supplementary Information.

## Data Availability

All relevant data, which supports the findings of the study, are within the manuscript.
