# Supplementary material for: HOPS, CORVET and newly-identified Hybrid tethering complexes contribute differentially towards multiple modes of endocytosis
Source: Sci Rep. 2023 Oct 31;13:18734. doi: 10.1038/s41598-023-45418-3 (PMC10618185; doi:10.1038/s41598-023-45418-3)

Supplementary Figure 1

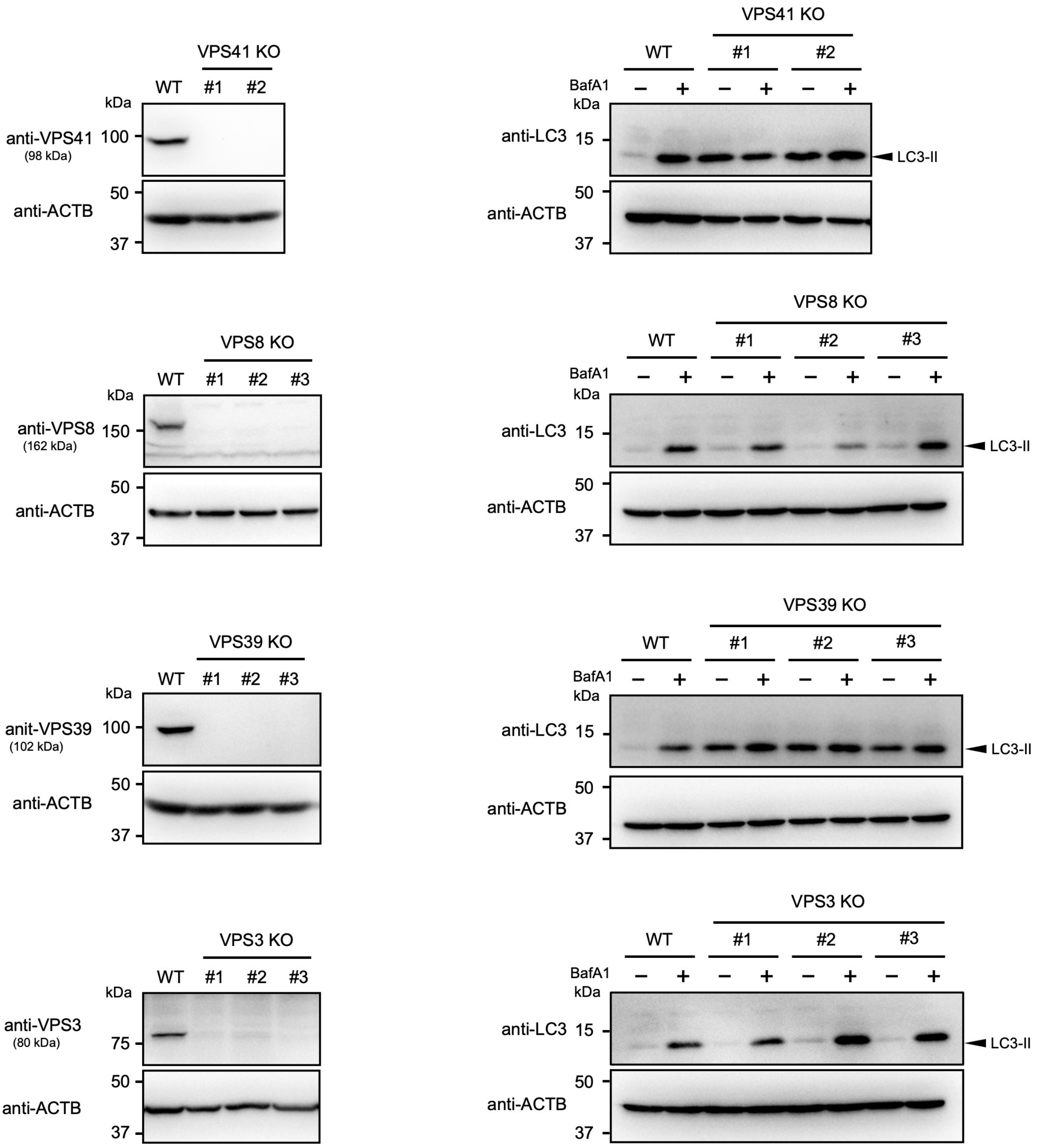

Characterisation of VPS single KO clones

The loss of protein expression (left side) and the phenotypic consistency of LC3 flux among the sub-clones (right side) of each VPS KO cell line were confirmed by western blotting.

Supplementary Figure 2

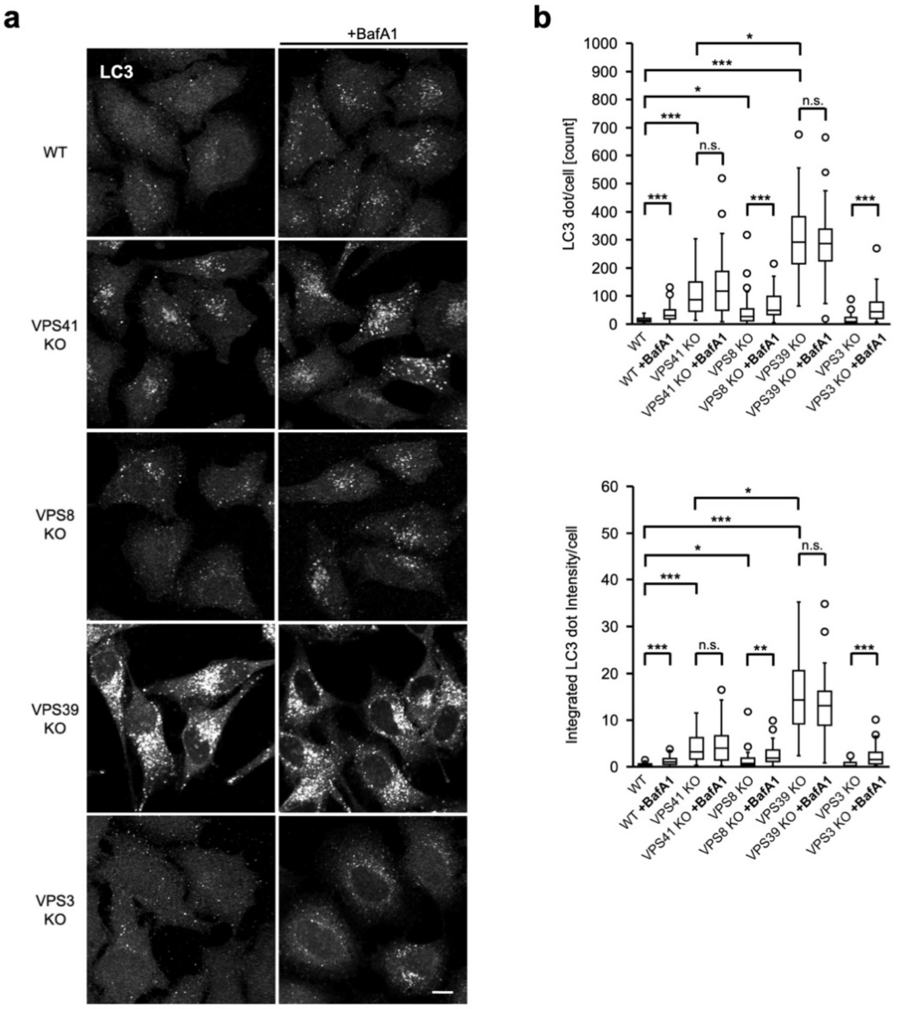

LC3 dot flux assay of VPS KO cells

a. Immunofluorescent analysis of LC3 in WT and VPS KO cells. Representative confocal images were shown. *Scale bar = 10 µm*. **b.** Quantitative analysis of LC3 dot counts and intensities in panel (a). Five images for each cell were captured. The LC3 dot number and integrated LC3 intensity for each cell were analysed by Cell Profiler™. LC3 dot number/cell (upper graph) and integrated LC3 dot intensity/cell (lower graph) are shown as a box-and-whisker plot. The counted cells for non-treated and BafA1 treated (BafA1) in WT; n = 59 and n = 89, VPS41 KO; n = 60 and n = 56, VPS8 KO; n = 55 and n = 49, VPS39 KO; n = 42 and n = 39, VPS3 KO; n = 57 and n = 46, respectively. Statistics were calculated by the Kruskal-Wallis test and followed by the Mann-Whitney U test with Holm correction. * $p < 0.05$, ** $p < 0.01$, *** $p < 0.001$, and n.s.; not significant.

Supplementary Figure 3

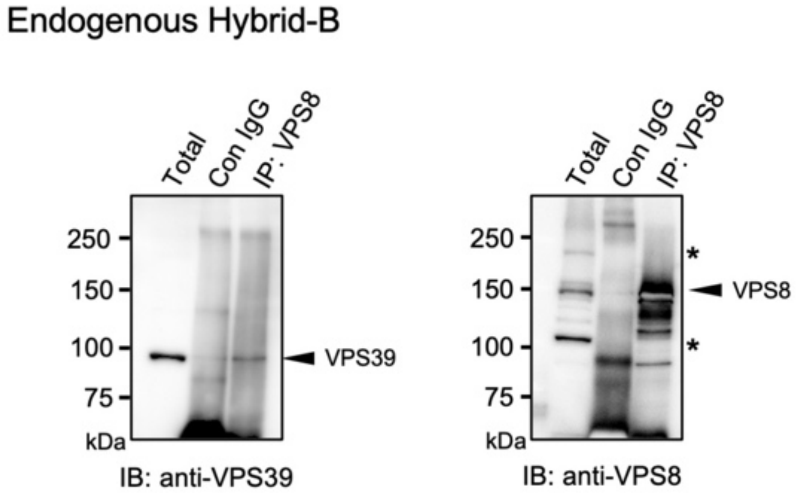

Detection of completely endogenous Hybrid-B complex by VPS8-IP

VPS8 was immunoprecipitated from extracts of WT HeLa cells, and VPS39 was detected from the immunoprecipitates by western blotting (left panel). Immunoprecipitation was confirmed using a VPS8 antibody; the VPS8 signal was too strong and partially burned out (right panel). Arrow heads indicate main band. Asterisks (*) represent non-specific bands.

Supplementary Figure 4

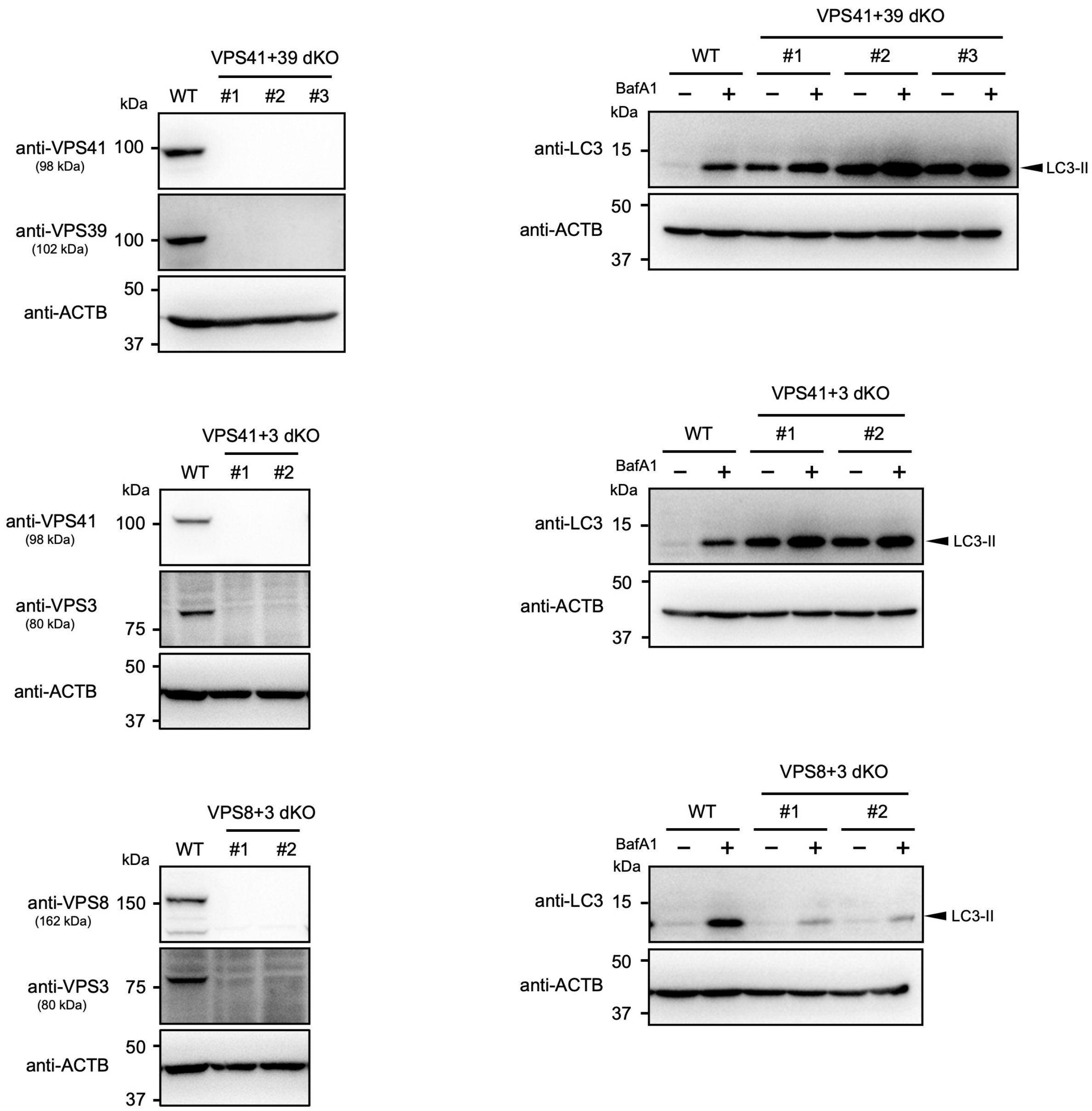

Characterisation of VPS double KO clones

The loss of protein expression (left panels) and the phenotypic consistency of LC3 flux among the sub-clones (right panels) in VPS dKO cells were confirmed by western blotting.

Supplementary Figure 5

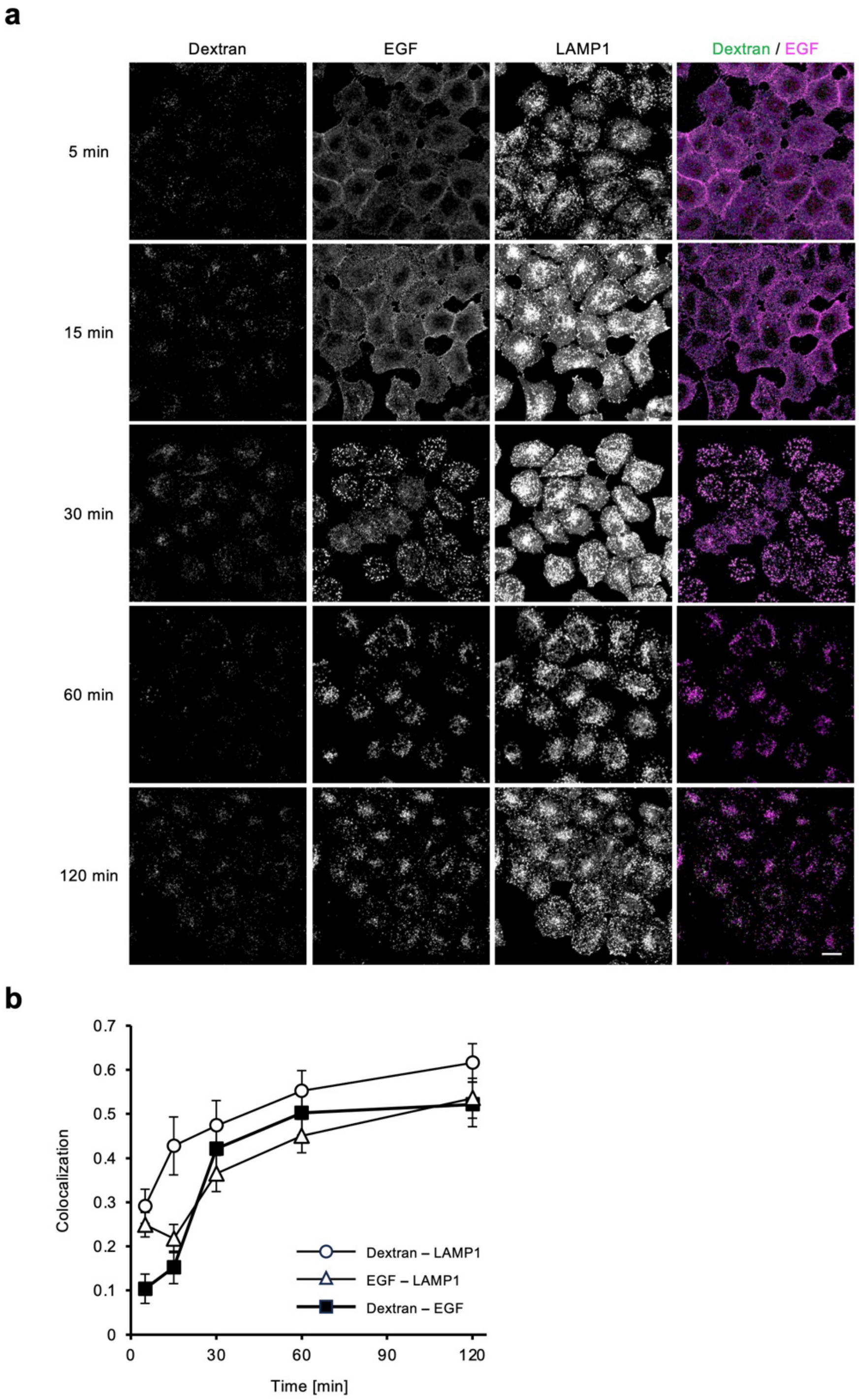

Time course tracking of two different types of endocytic cargo in WT cells

a. WT HeLa cells were simultaneously treated with Alexa Fluor 488-labelled dextran and Alexa Fluor 647-labelled EGF for the indicated time. Each cell was fixed and fluorescently stained with an anti-LAMP1 antibody. Representative confocal microscopy images at each

time point are shown. *Scale bar = 10 µm*. **b.** Colocalisation of dextran with LAMP1, EGF with LAMP1, and LAMP1 with EGF at each time point is shown as Pearson's correlation coefficient ± S.D.

Supplementary Table 1

Gene name	**Sequence of crRNA+PAM in guide RNA (5' to 3')**
VPS8	CTCCTATGATACTTCATCTGTGG
VPS41	TACACCTATGACAAGAACTATGG<br>TGCTACTGTTTGAACGGTCTTGG
VPS3	CTTAGACGAGGAACTAGTGTGGG
VPS39	TCTTCTCTATAGGATTCGGAAGG

crRNA sequence used for the generation of knockout cell lines

The knockout cell lines were established by CRISPR/Cas9 system. Target genes and sequences of crRNA used for the establishment of the knockout cells are shown. The PAM sequence for each is indicated with the underline.

Supplementary Table 2

Cell line	*Gene*	Mutation	
		Allele 1	Allele 2
VPS8 KO #1*	*VPS8*	ins 1 bp	del 1 bp
VPS8 KO #2		ins 1 bp	ins 2 bp
VPS8 KO #3		ins 1 bp	homo
VPS41 KO #1*	*VPS41*	del 1 bp	del 2 bp
VPS41 KO #2		del 2 bp	del 14 bp
VPS3 KO #1*	*VPS3*	del 11 bp	del 16 bp
VPS3 KO #2		del 102 bp	del 4 bp
VPS3 KO #3		ins 1 bp	del 28 bp
VPS39 KO #1*	*VPS39*	ins 1 bp	del 1 bp
VPS39 KO #2		ins 1 bp	del 8 bp
VPS39 KO #3		ins 1 bp	homo
VPS8+VPS3 dKO #1*	*VPS8, VPS3*	*VPS8*: ins 1 bp<br>*VPS3*: del 1 bp	*VPS8*: del 1 bp<br>*VPS3*: del 17 bp
VPS8+VPS3 dKO #2		*VPS8*: ins 1 bp<br>*VPS3*: ins 2 bp	*VPS8*: del 1 bp<br>*VPS3*: del 5 bp
VPS41+VPS3 dKO #1*	*VPS41, VPS3*	*VPS41*: del 1 bp<br>*VPS3*: del 29 bp	*VPS41*: del 2 bp<br>*VPS3*: del 134 bp
VPS41+VPS3 dKO #2		*VPS41*: del 1 bp<br>*VPS3*: del 11 bp	*VPS41*: del 2 bp<br>*VPS3*: del 121 bp
VPS41+VPS39 dKO #1*	*VPS41, VPS39*	*VPS41*: del 1 bp<br>*VPS39*: ins 1 bp	*VPS41*: del 2 bp<br>*VPS39*: homo
VPS41+VPS39 dKO #2		*VPS41*: del 1 bp<br>*VPS39*: ins 1 bp	*VPS41*: del 2 bp<br>*VPS39*: homo
VPS41+VPS39 dKO #3		*VPS41*: del 1 bp<br>*VPS39*: ins 1 bp	*VPS41*: del 2 bp<br>*VPS39*: homo

Genotypes of cell lines used in the present study

The knockout cell lines were established by CRISPR/Cas9 technique. Target genes are shown with the mutations introduced by gene targeting. Asterisks (*) represent the clones mainly used in the figures.

Full Membrane Blots

Figure 1

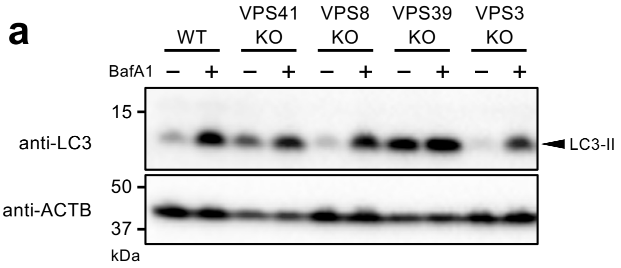

anti-LC3 Original

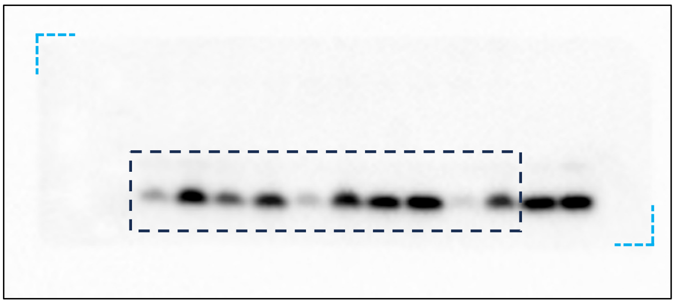

anti-ACTB Original

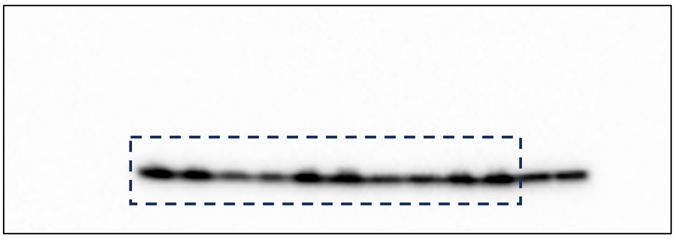

anti-ACTB + Membrane

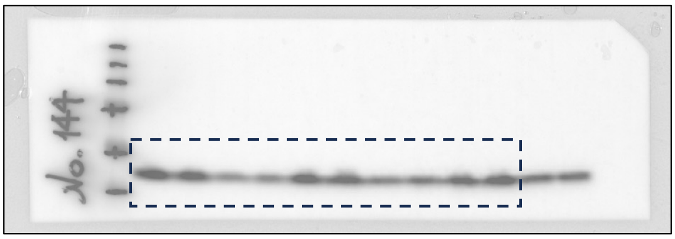

Full Membrane Blots

Figure 1

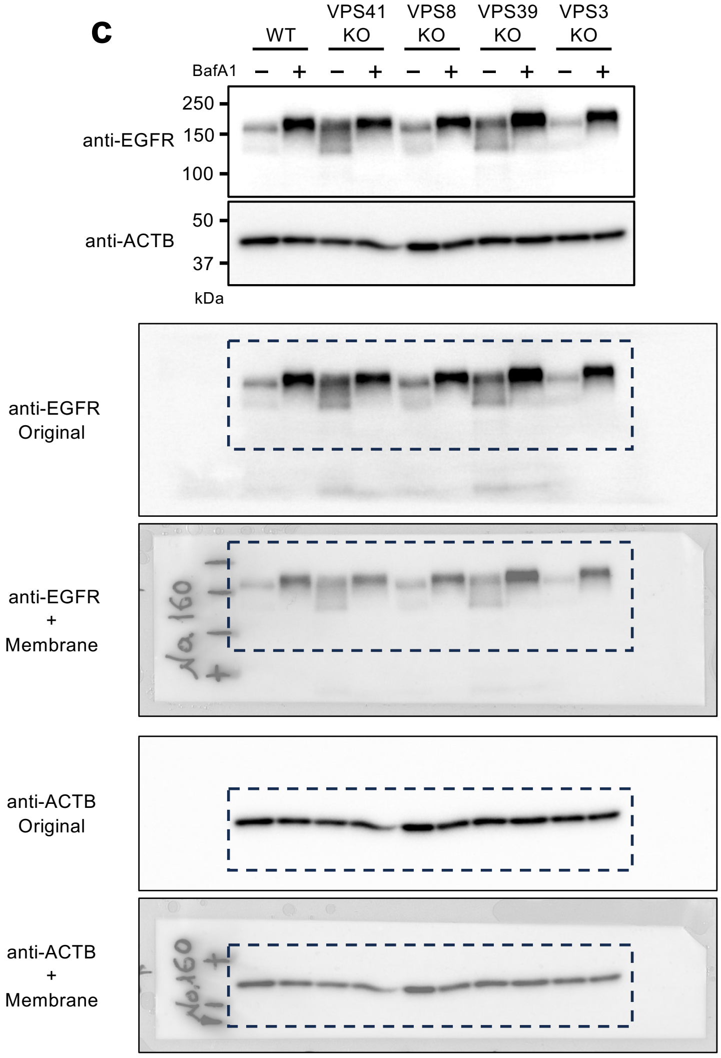

Full Membrane Blots

Figure 2

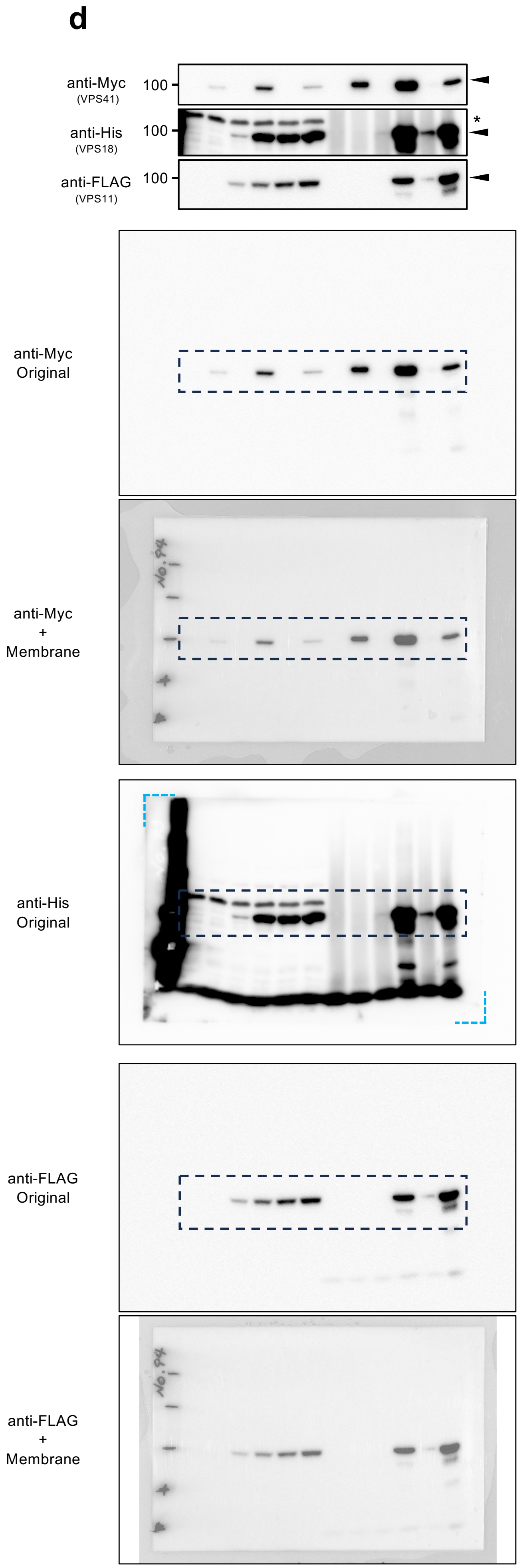

Full Membrane Blots

Figure 2

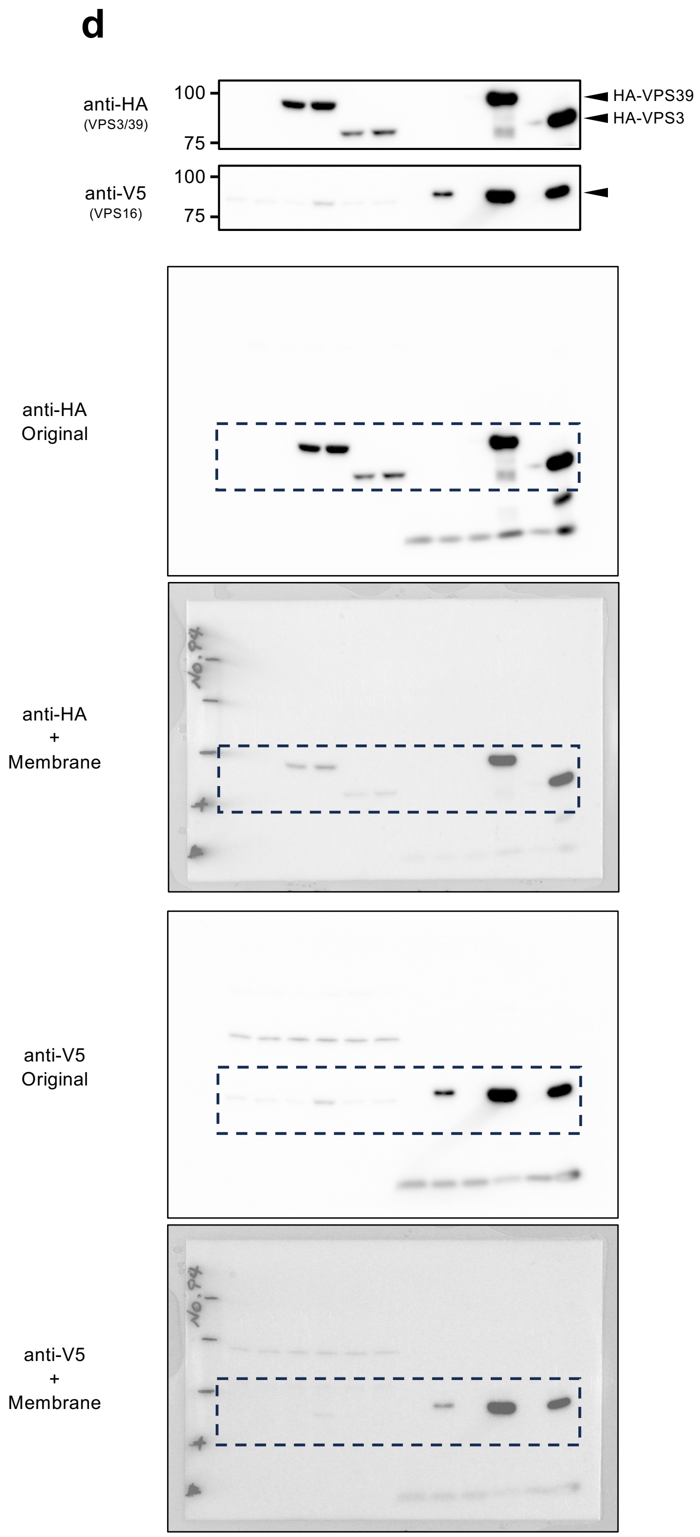

Full Membrane Blots

Figure 2

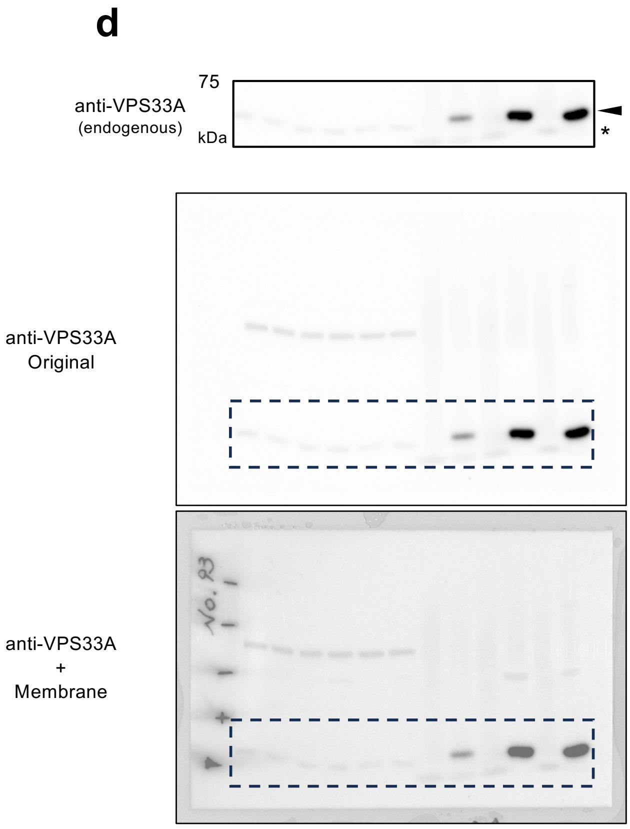

Full Membrane Blots

Figure 2

e

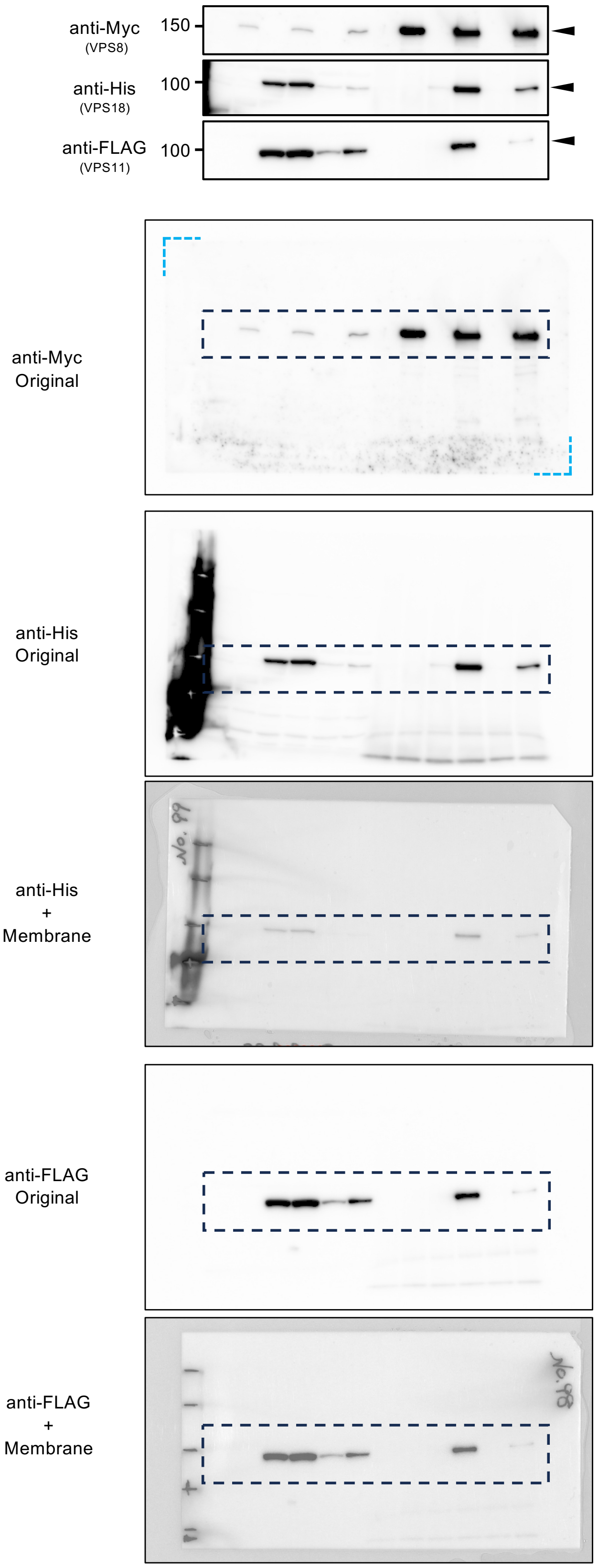

Full Membrane Blots

Figure 2

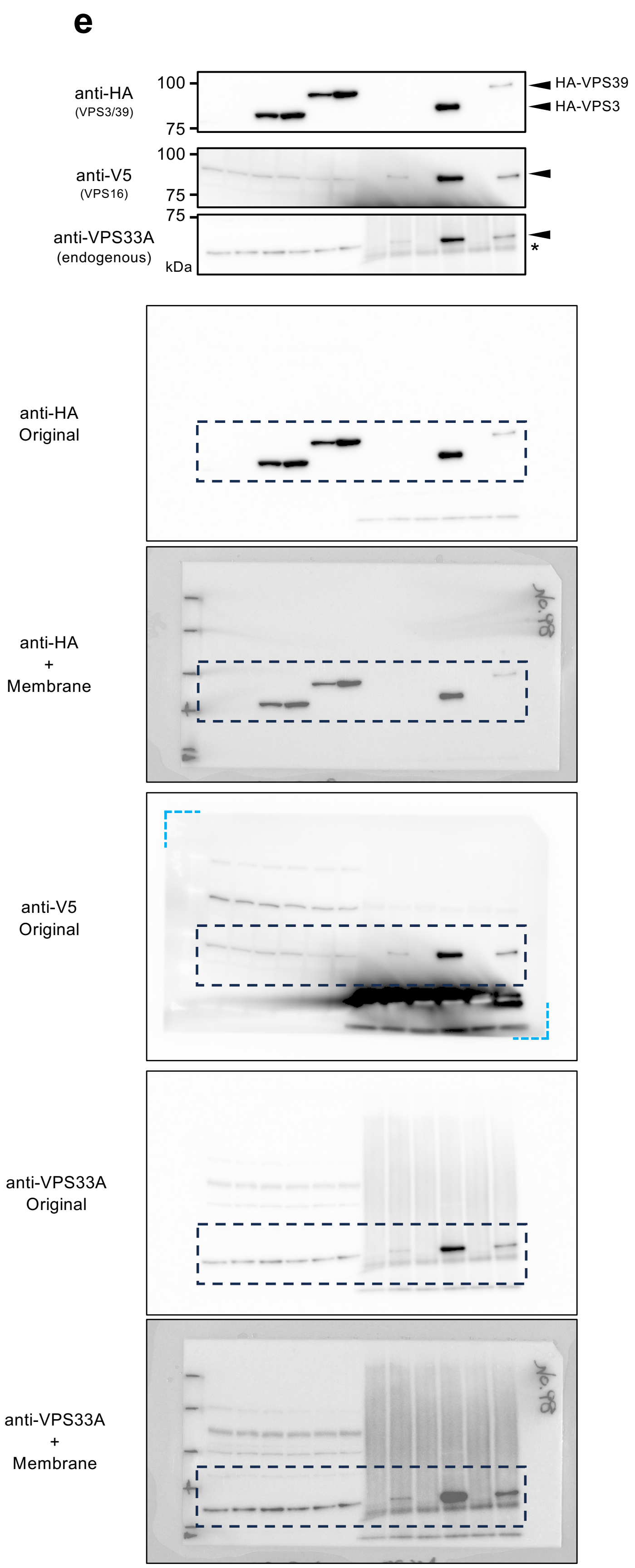

Full Membrane Blots

Figure 3

b

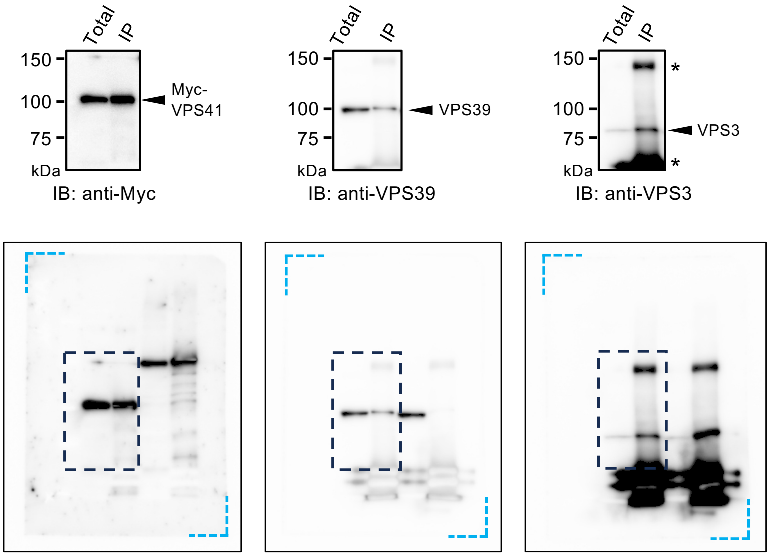

Original

c

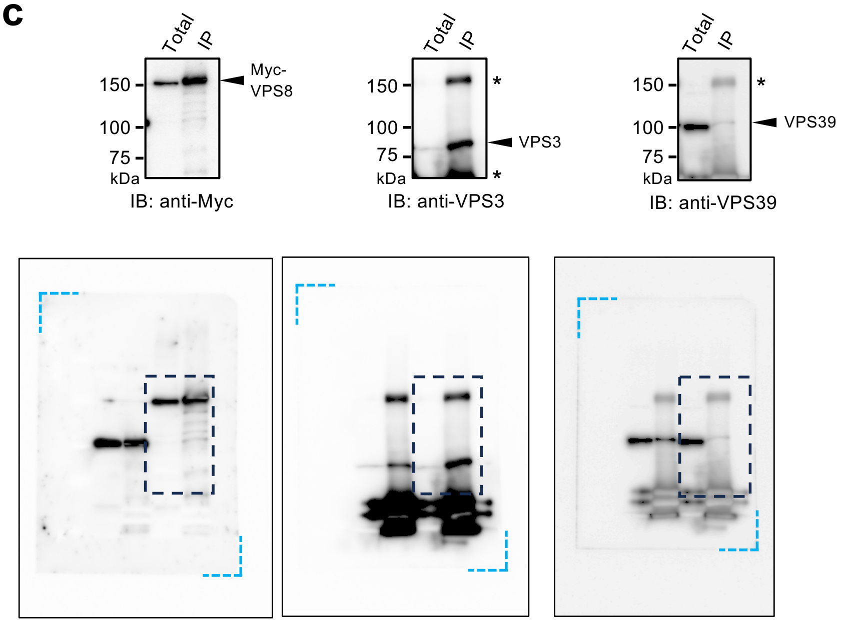

Original

Full Membrane Blots

Figure 3

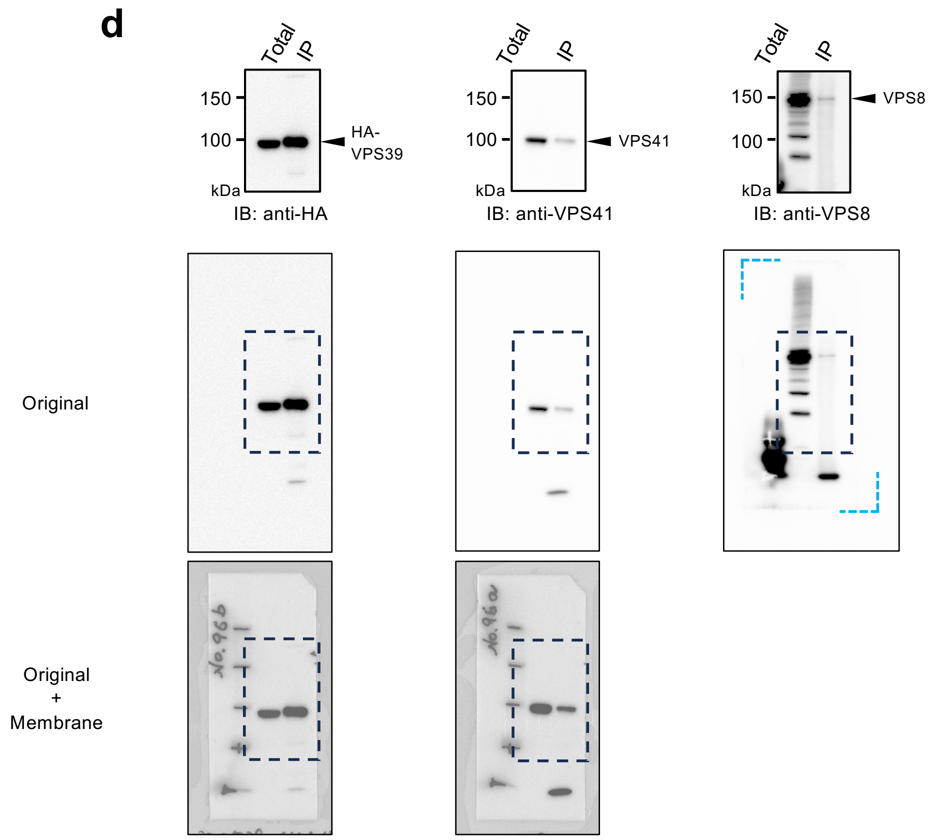

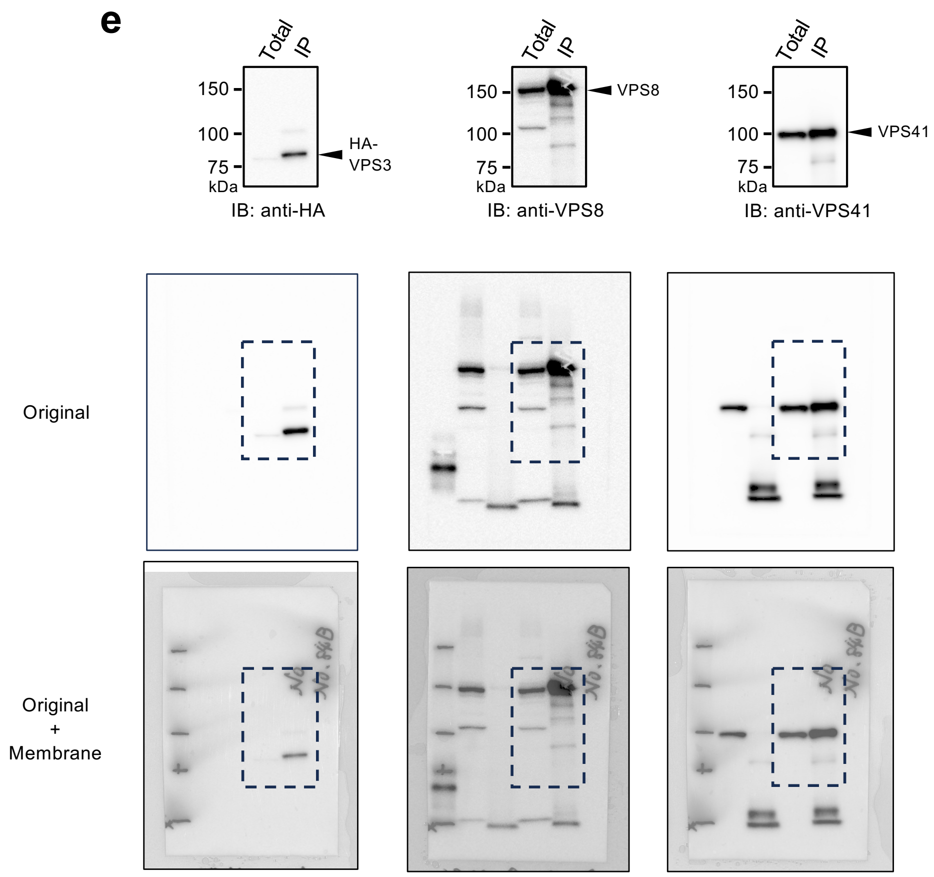

Full Membrane Blots

Figure 4

a

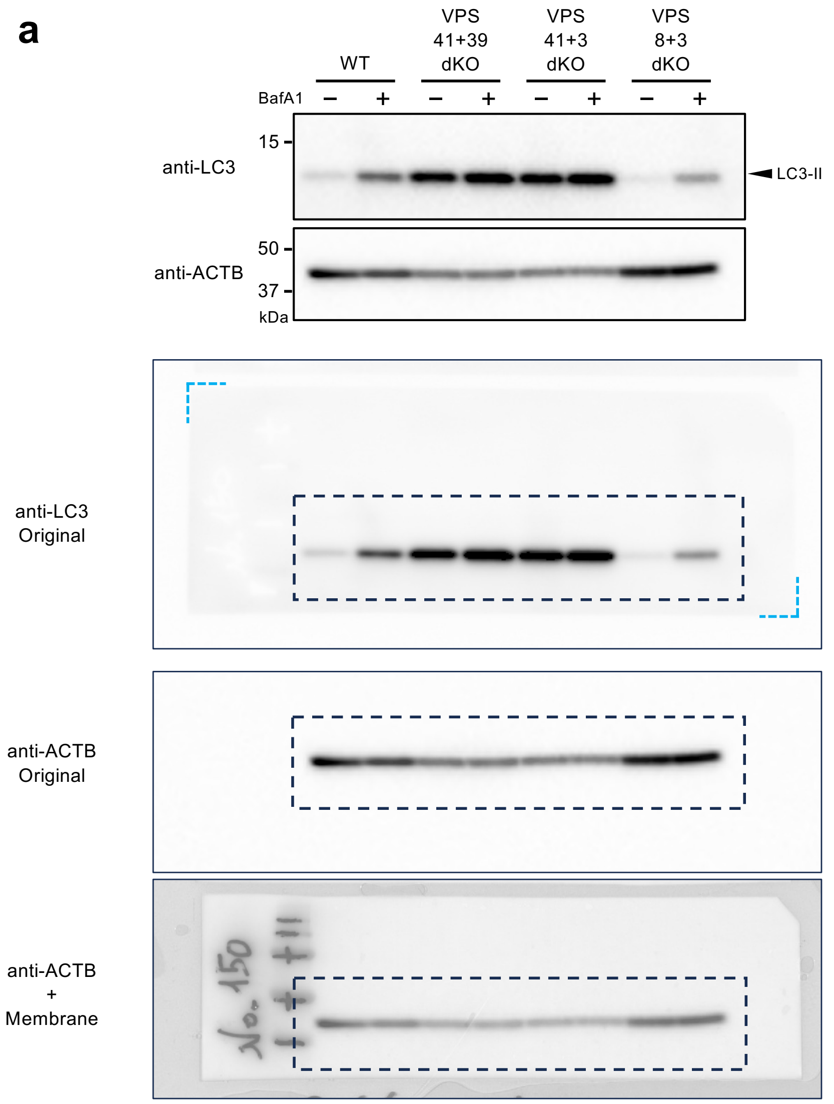

Full Membrane Blots

Figure 4

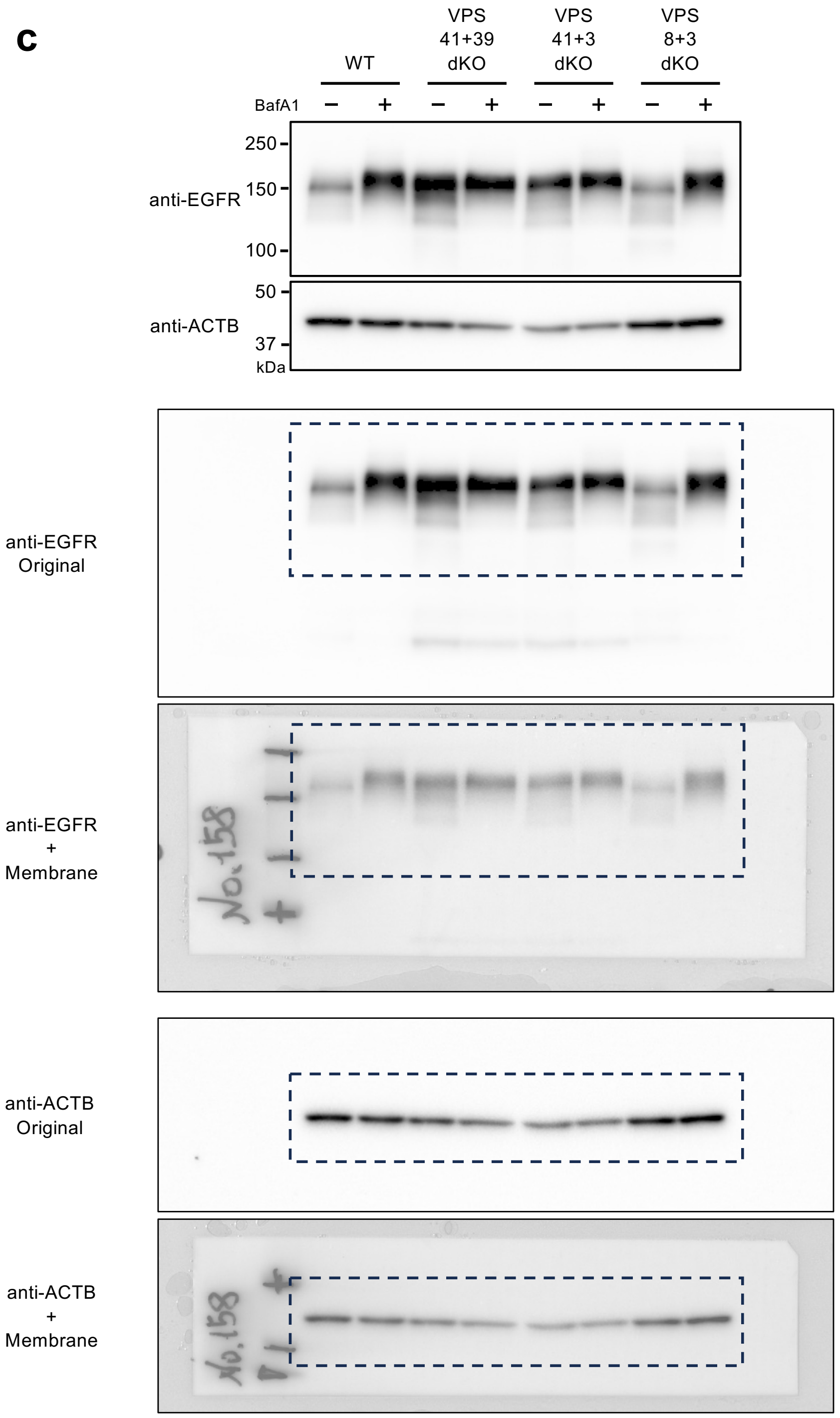

Full Membrane Blots

Supplementary Figure 1

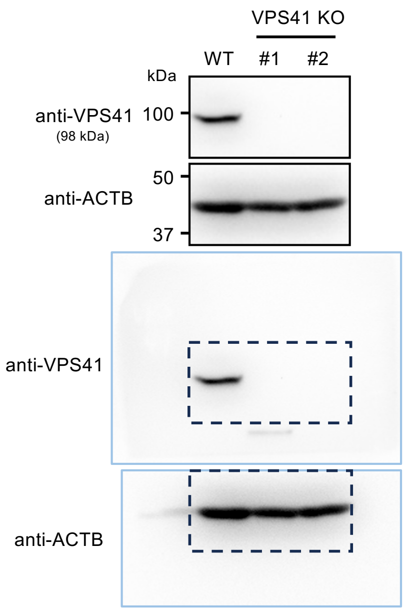

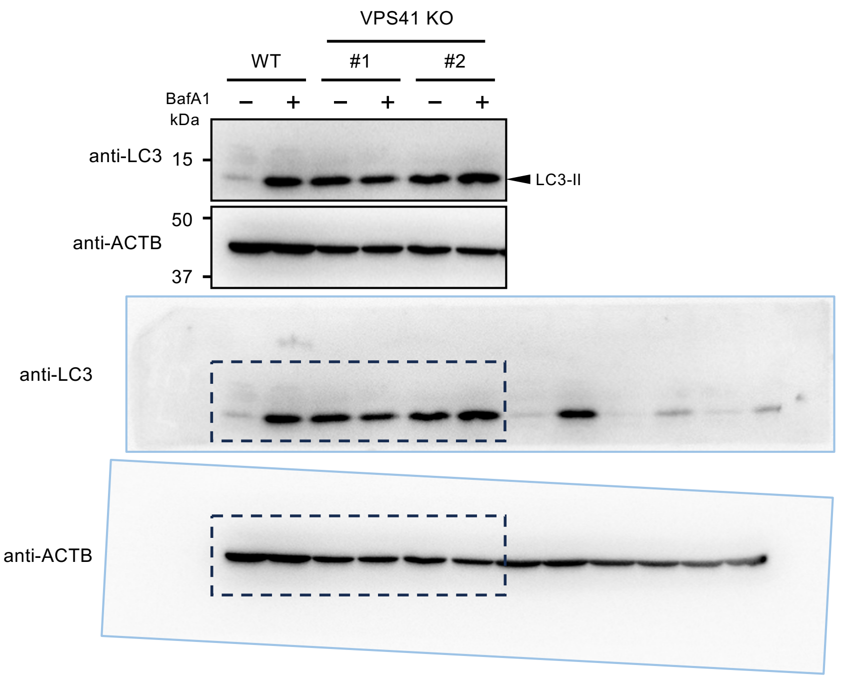

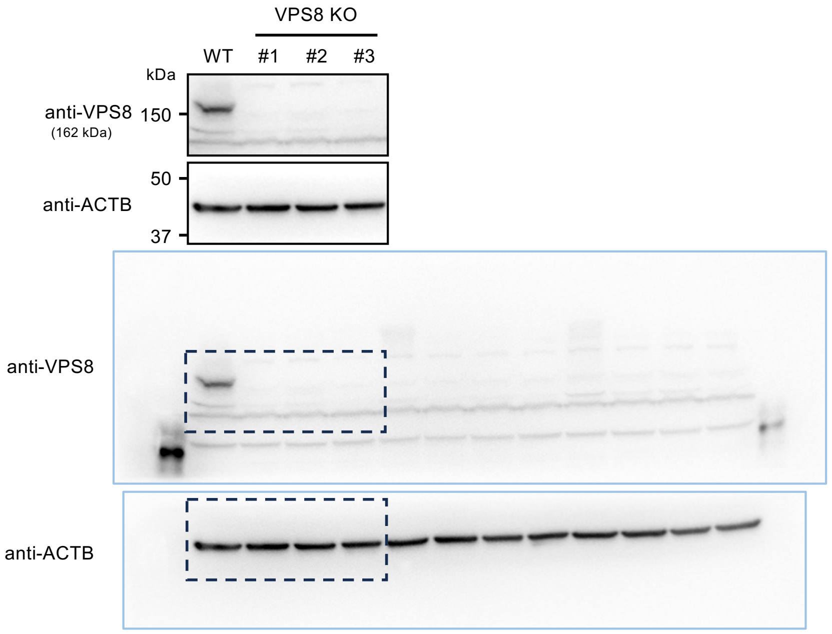

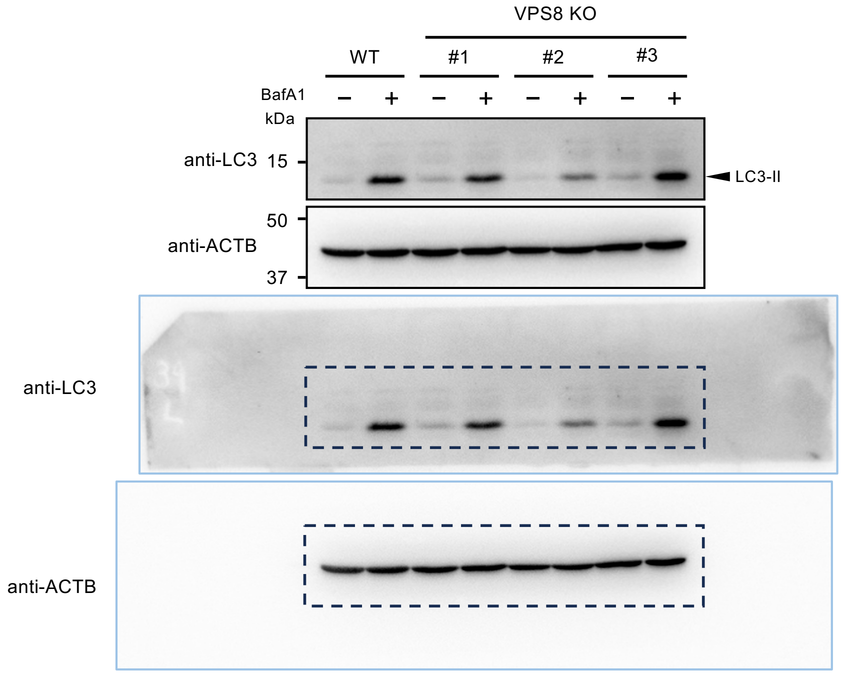

Full Membrane Blots

Supplementary Figure 1

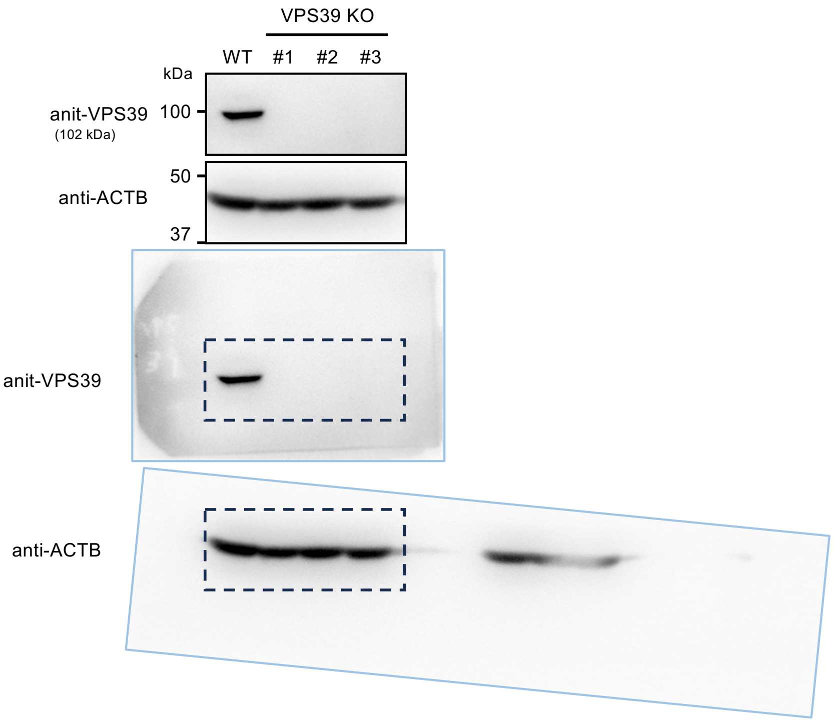

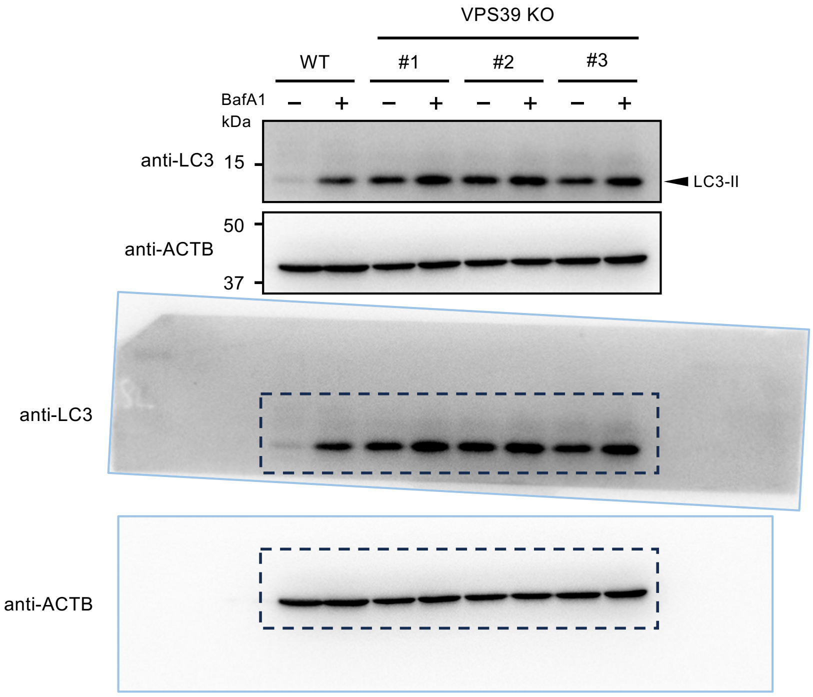

Full Membrane Blots

Supplementary Figure 1

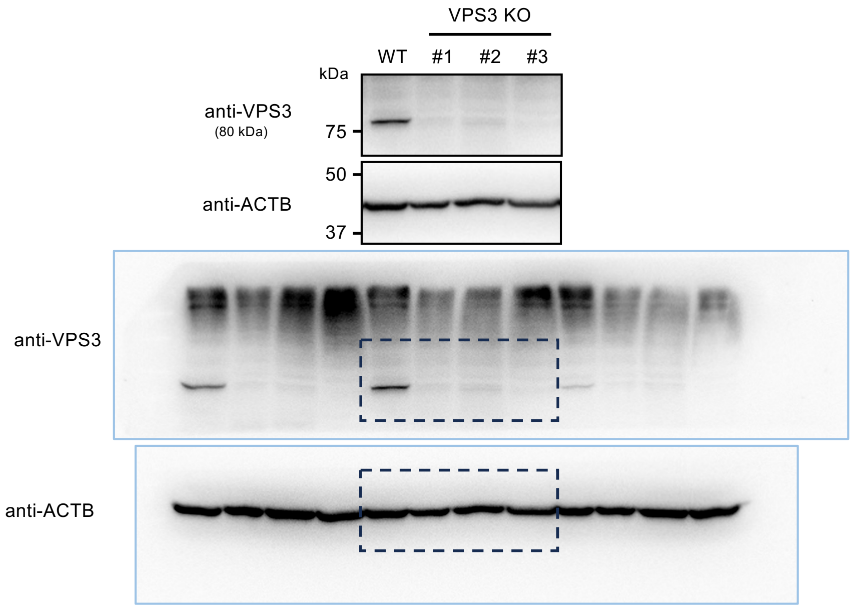

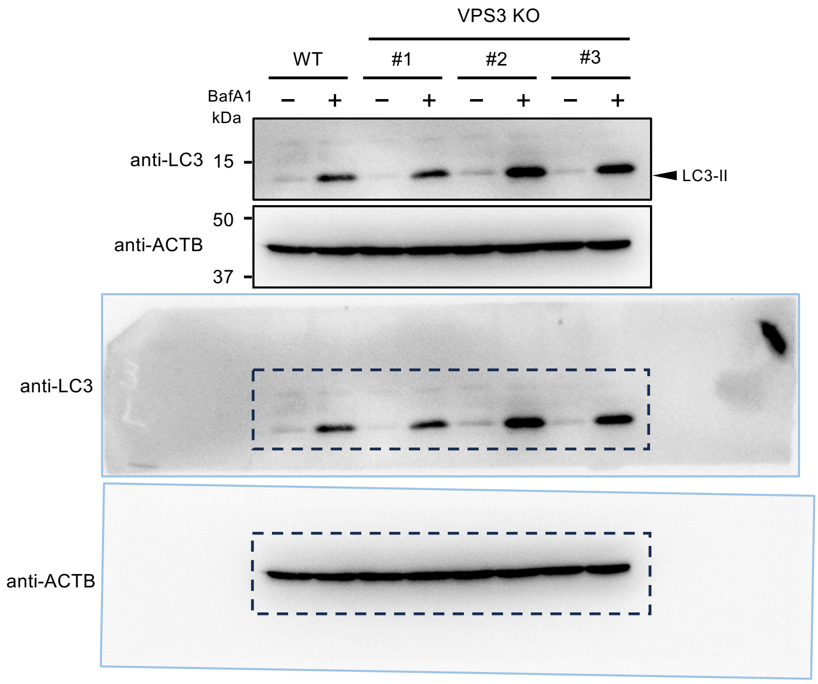

Full Membrane Blots

Supplementary Figure 3

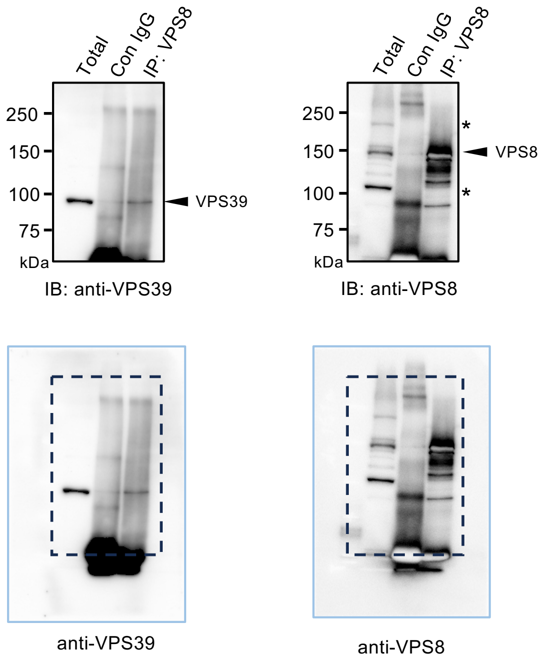

Full Membrane Blots

Supplementary Figure 4

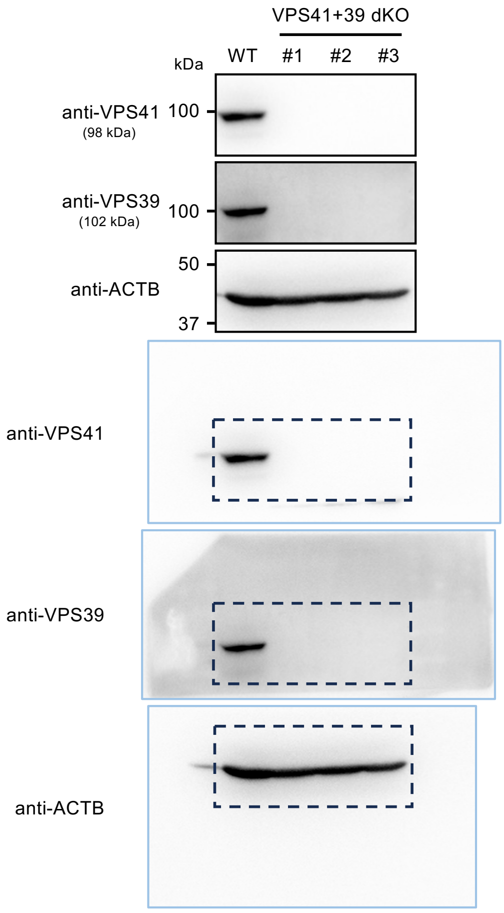

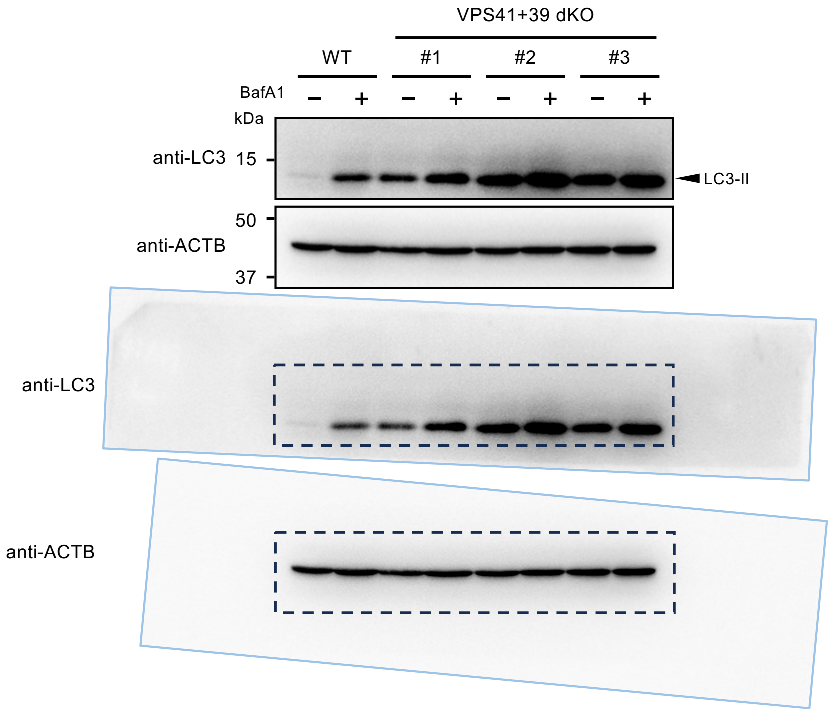

Full Membrane Blots

Supplementary Figure 4

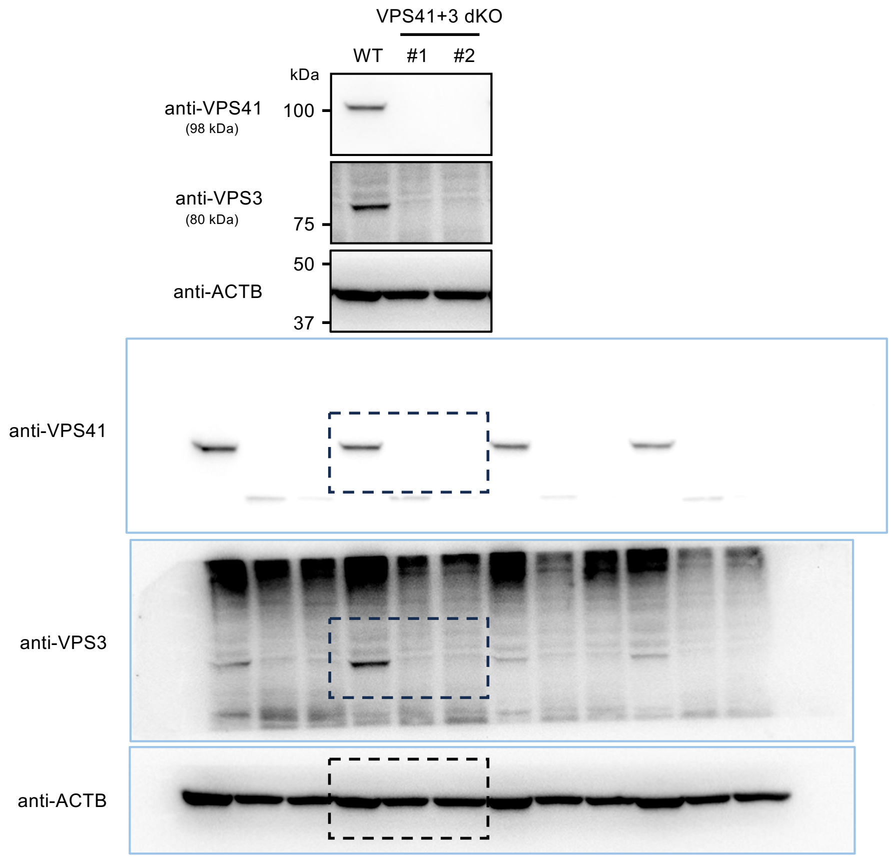

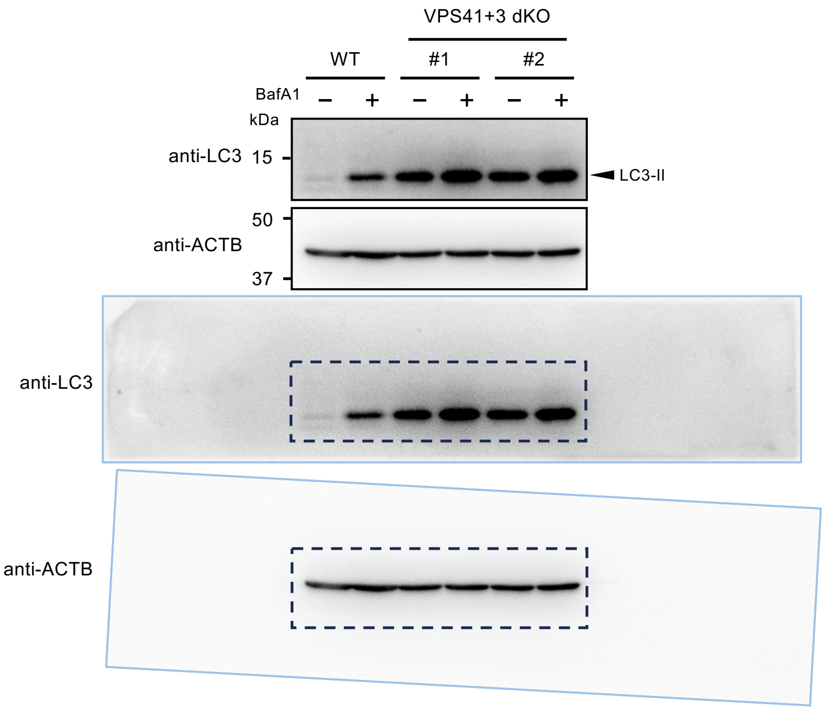

Full Membrane Blots

Supplementary Figure 4

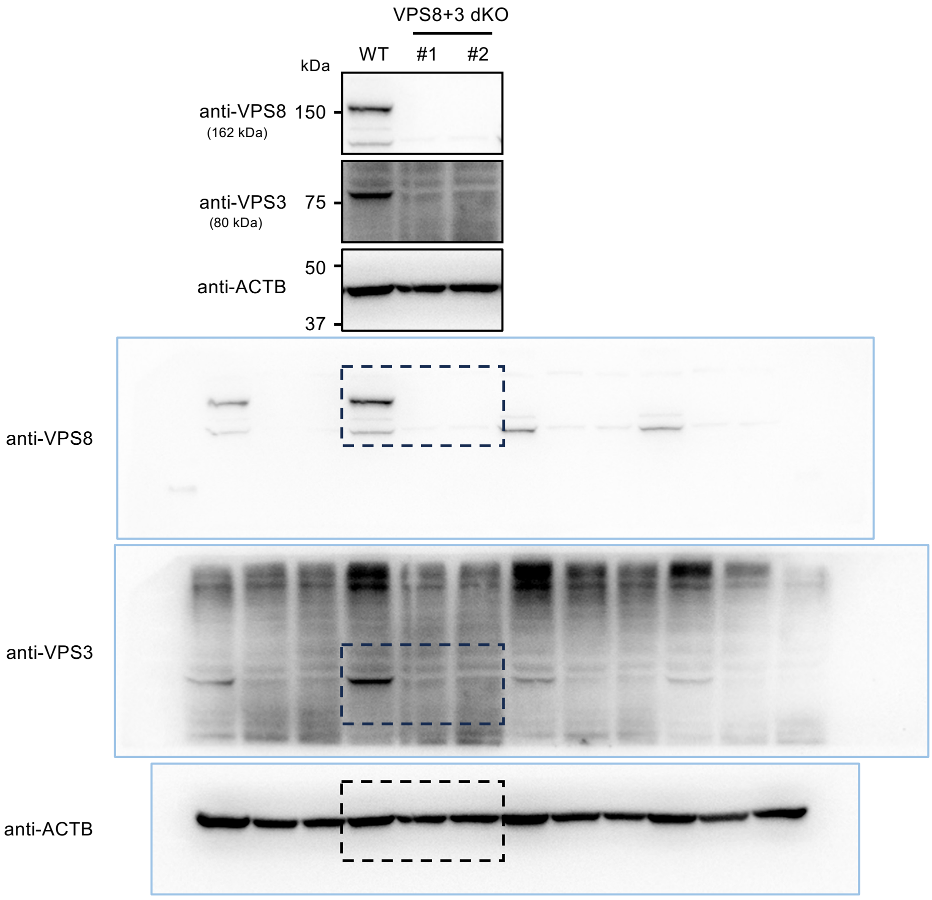

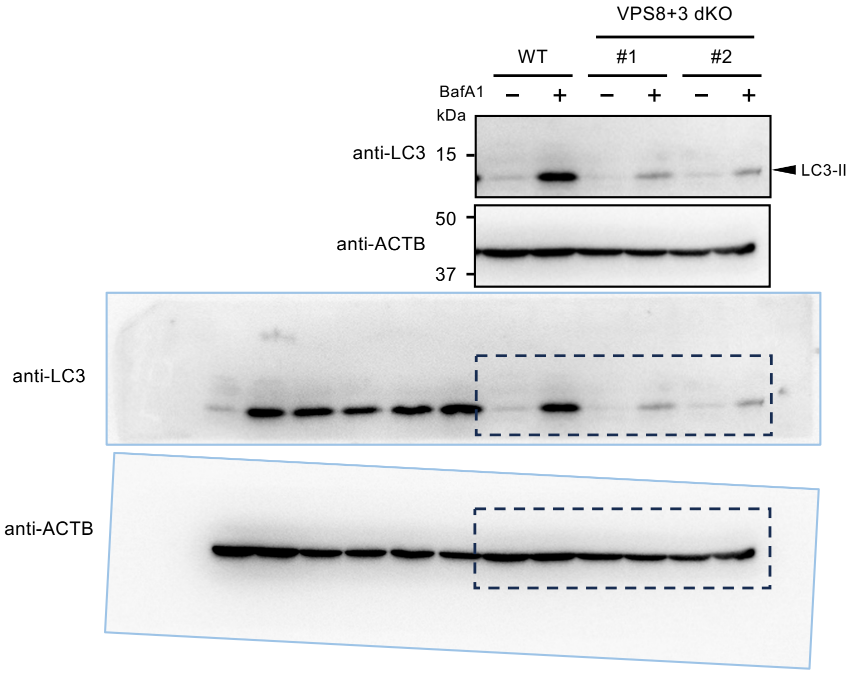

Supplement: Supplementary file 1 — Supplementary Information. [file 41598_2023_45418_MOESM1_ESM.pdf]
